# Climate change-related warming reduces thermal sensitivity and modifies metabolic activity of coastal benthic bacterial communities

**DOI:** 10.1038/s41396-023-01395-z

**Published:** 2023-03-28

**Authors:** Laura Seidel, Elias Broman, Emelie Nilsson, Magnus Ståhle, Marcelo Ketzer, Clara Pérez-Martínez, Stephanie Turner, Samuel Hylander, Jarone Pinhassi, Anders Forsman, Mark Dopson

**Affiliations:** 1 Centre for ecology and evolution in microbial model systems (EEMiS), Linnaeus University, Kalmar, Sweden; 2 Department of Ecology, Environment and Plant Sciences, Stockholm University, Stockholm, Sweden; 3 Baltic Sea Centre, Stockholm University, Stockholm, Sweden

**Keywords:** Water microbiology, Climate-change ecology, Transcriptomics, Microbial ecology, Climate-change impacts

## Abstract

Besides long-term average temperature increases, climate change is projected to result in a higher frequency of marine heatwaves. Coastal zones are some of the most productive and vulnerable ecosystems, with many stretches already under anthropogenic pressure. Microorganisms in coastal areas are central to marine energy and nutrient cycling and therefore, it is important to understand how climate change will alter these ecosystems. Using a long-term heated bay (warmed for 50 years) in comparison with an unaffected adjacent control bay and an experimental short-term thermal (9 days at 6–35 °C) incubation experiment, this study provides new insights into how coastal benthic water and surface sediment bacterial communities respond to temperature change. Benthic bacterial communities in the two bays reacted differently to temperature increases with productivity in the heated bay having a broader thermal tolerance compared with that in the control bay. Furthermore, the transcriptional analysis showed that the heated bay benthic bacteria had higher transcript numbers related to energy metabolism and stress compared to the control bay, while short-term elevated temperatures in the control bay incubation experiment induced a transcript response resembling that observed in the heated bay field conditions. In contrast, a reciprocal response was not observed for the heated bay community RNA transcripts exposed to lower temperatures indicating a potential tipping point in community response may have been reached. In summary, long-term warming modulates the performance, productivity, and resilience of bacterial communities in response to warming.

## Introduction

Climate change is an ongoing threat that is projected to cause severe effects on the Earth’s surface and oceans such as increasing temperatures [1] and more extreme weather events [2]. Greenhouse gases, such as CO_2_ and CH_4_, accumulate in the atmosphere resulting in increased surface temperatures and are also dissolved in the world´s oceans [3]. These combined effects lead to changes in marine salinity, acidification, stratification, deoxygenation, and increased microbial metabolic rates [4–6]. For example, long-term temperature observations of the upper 2000 m of the oceans show an average temperature increase of 0.09 °C since 1955 that is equivalent to an energy increase of 24 × 10^22 ^J yr^−1^ [7]. Furthermore, marine heatwaves are projected to increase on average by a factor of 16–41 from 1.5 to 3.5 °C of warming by the end of 2100 [2] that will result in further greenhouse gas emissions [8].

Marine heatwaves, location-specific extreme warm water events that occur for a prolonged period of time [9, 10], have occurred with increasing regularity and time span within the last century [11]. The increase in duration, intensity, and frequency of marine heatwaves is linked to human-induced climate change [2, 12] although to varying extents based on the location [9]. For example, the Baltic Sea has experienced a large temperature increase and higher frequency of marine heatwaves over the last decades, such as the 2014 Swedish heatwave [13] or the 2018 heatwave [14]. These warming trends affect not only the sea surface (0–2 m) but also the entire water column, such as seen within the south–west Finnish coastline that experienced 18 detected heatwaves on bottom waters (30 m depth) within four years of high-resolution recordings [15]. However, the rate and degree of this effect remains unknown.

Coastal areas are highly productive ecosystems [16, 17] while at the same time being among the most vulnerable regions worldwide [18, 19]. Microorganisms within these areas are central to the marine energy and nutrient cycles, acting as primary producers of biomass as well serving as degraders of organic material in the water column and on the sea floor [20]. Microorganisms are also important key players in the regulation of the production and fluxes of greenhouse gases such as methane [21], dimethyl sulfide [22], and nitrous oxide [23, 24]. Changes or shifts in microbial community composition as well as their ability to respond to environmental changes such as global warming, will have severe effects on marine geochemistry and ecosystem functioning. However, the manner in which microbial ecosystem functioning will be disturbed remains unknown.

Temperature influences virtually all aspects of organismal performance. Thermal biology seeks to understand contemporary variation and evolutionary modifications of such thermal sensitivity by quantifying and comparing the shape of the relationships (reaction norms) linking the rate or efficiency of functions to temperature [25, 26]. It is widely recognized that the thermal performance curves of individuals, populations, and species can change owing to the combined effects of phenotypic plasticity and evolutionary modifications of physiological processes [26]. In theory, the sum of such intraspecific changes should have the potential to also alter the thermal performance curves of community processes. Furthermore, both the location of the optima and breadth of the thermal performance curve of a community can change if there are shifts in the composition or relative abundances of species with different thermal sensitivities [27, 28]. However, it is unknown how the many ways by which climate change will affect the thermal performance of communities and the ecosystem services that they provide, such as bacterial production.

Experimental investigations of the trade-off between the peak production (height) and the temperature tolerance (breadth) of the thermal performance curves seen within species [25, 26, 29] have not been extensively investigated at the community level. This is especially true during long-term warming itself or in combination with short-term marine heatwave events, yet such information is key to project the consequences of future climate change warming for ecosystem functioning [30].

In this study, the goal was to investigate the thermal responses to short-term, high-intensity temperature increases (such as marine heatwaves) of coastal marine microbial communities by comparing geochemical parameters, bacterial production, 16 S rRNA gene amplicons, and community RNA sequencing on bottom water and surface sediment of a long-term heated bay (warmed for 50 years) in comparison with a control bay [31]. We hypothesized that temperature changes such as occurring during short-term marine heatwaves would result in the control bay bacterial community becoming more similar to the heated bay response. More specifically, the following questions were investigated: (1) Do the thermal performance curves differ between the heated and non-heated (control) bays? (2) Does short-term temperature increase, such as occurring in marine heatwaves, alter the bacterial community composition and/or metabolism in the heated and control bays differently? (3) Can already altered long-term warmed bacterial communities go back to a contemporary state, such as seen in the control bay? To answer these questions, a laboratory thermal incubation experiment (mesocosm) was performed at a range of temperatures between 6 and 35 °C (6, 8, 15, 16, 24, 25, 28, and 35 °C) that was similar to the annual temperature range of the studied marine coastal bays.

## Results

### Chemical measurements in response to temperature incubation

**Fig. 1:**
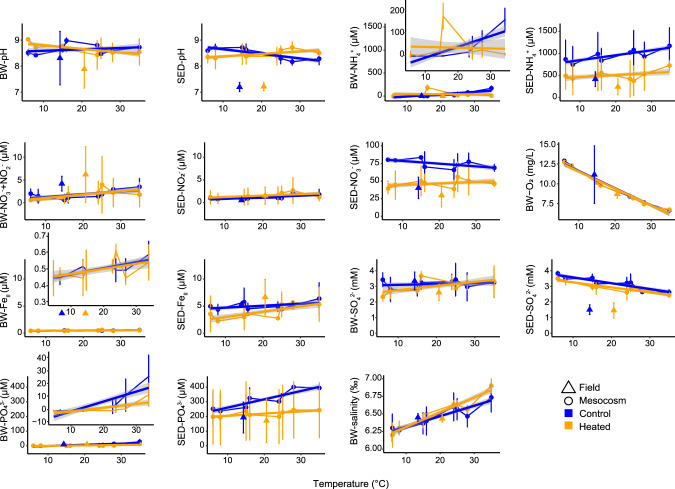
Geochemical parameters of the heated (orange) and control (blue) in bottom and pore waters. The parameters were measured in the bottom water and sediment porewater (0–1 cm) of the incubated cores between 6 and 35 °C after 9 days of incubation. A total of *n* = 3 samples per bay per temperature for bottom water (*n* = 48) and porewater (*n* = 48) were collected. Each point shows the mean of *n* = 3 cores per bay plus error bar shows the standard deviation. Regression lines are presented to follow temperature-related patterns.

At the time of sampling, the mean (*n* = 3) bottom water temperature in the field heated bay was 20.6 ± 0.3 °C while it was 14.3 ± 1.9 °C for the control bay and the mesocosm incubation temperatures ranged from 6–35 °C. The annual mean water temperature in 2018 was 18.3 ± 5.9 °C in the heated and 13.9 ± 7.3 °C in the control bay [31]. Several chemical parameters in bottom water and sediment changed along the experimental temperature treatment gradient (Fig. 1 and Supplementary Figs. S1 and S2). Bottom water mesocosm analysis showed significantly decreasing oxygen concentration with elevated temperatures for both bays (temperature gradient: 6–35 °C, ANOVA, *F*_9,38_ = 218.3, *P* < 0.001; Fig. 1, Supplementary Fig. S1, and Supplementary Table S1). The combination of nitrate + nitrite (*F*_9,38_ = 8.2, *P* = 0.006), ferrous iron (*F*_9,38_ = 7.8, *P* = 0.007), and phosphate (*F*_9,38_ = 49.6, *P* < 0.001; Supplementary Tables S1 and S2) showed significant increasing bottom water concentrations with increasing temperatures in both mesocosms from the bays. Finally, we did not observe any changes in the water volume, and the increase in salinity may have been due to changes in the solubility of salts and exchange between the water column and sediment.

Surface sediment porewater (top 0–1 cm sediment) ammonium showed lower concentrations in the heated bay porewater field data (240.9 ± 180.6 µM; Supplementary Table S2) that continued to be significantly lower (491.2 ± 311.5 µM) in the incubations compared to the control bay mesocosms (724.4 ± 113.2 µM; ANOVA, *F*_9,38_ = 88.5, *P* < 0.001; Supplementary Tables S1 and S2). Sediment phosphate was also significantly different between the two bays mesocosms (*F*_9,38_ = 69.8, *P* < 0.001) with increasing concentrations in both bays with rising temperature (effect of temperature, *F*_9,38_ = 34.7, *P* < 0.001; Supplementary Fig. 1 and Supplementary Tables S1 and S2). Porewater nitrate concentrations were overall lower in the heated bay field and incubation cores (Fig. 1 and Supplementary Table S2). In detail, nitrate showed a 39.4 ± 7.8 µM to 46.2 ± 4.0 µM increase with elevated temperatures, while an 80.3 ± 30.6 µM to 68.9 ± 5.1 µM decreasing trend was observed in the control bay incubations (Fig. 1 and Supplementary Table S2) showing significant differences between the bays (*F*_9,38_ = 95.1, *P* < 0.001; Supplementary Table S1). Porewater sulfate concentrations showed a significant decreasing trend in both bays mesocosms with elevated temperatures (effect of temperature, *F*_9,38_ = 77.1, *P* < 0.001; Fig. 1 and Supplementary Tables S1 and S2).

### Bacterial production at different temperatures

**Fig. 2:**
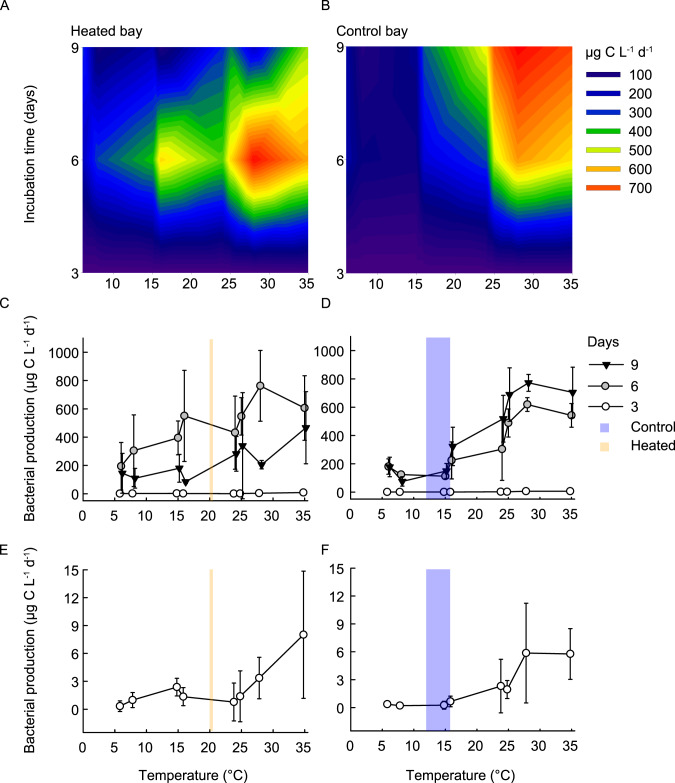
Bacterial production in the bottom water of the heated and control bays at the different incubation temperatures. Surface plot of the bacterial production (in µg carbon per liter per day) in the heated (**A**) and control (**B**) bays after 3, 6, and 9 days of incubation at the different temperatures between 6 and 35 °C. In addition, an overview over the bacterial production (in µg carbon per liter per day as linear scale) for each time point and incubation temperatures in the heated (**C**) and control (**D**) bay; shown are the mean ± s.d. (*n* = 3 per bay) for each incubated temperature (6–35 °C) after 3 (white circle), 6 (gray circle), and 9 (black triangle) days of incubation. Furthermore, incubation day 3 is shown separately in the bottom panels (**E**) and (**F**) to gain a better visualization of the data. Finally, the blue (control) and orange (heated) shaded areas in (**C**–**F**) indicate the temperature range measured in the field at the time of sampling (time point zero).

The shape of the thermal performance curves linking bacterial production in bottom water to incubation temperature differed between the bays and changed over time (as evidenced by the three-way interaction between bay, time, and temperature; *F*_2,121_ = 3.5, *P* = 0.032; Fig. 2 and Supplementary Table S1). Overall, the thermal performance curves were flatter (i.e., indicative of broader thermal tolerance) in the heated than in the control bay mesocosms (Fig. 2 and Supplementary Fig. S3). The highest bacterial production values were measured at 28 °C after nine (mean ± s.d., 772.4 ± 59.9 µg C L^−1^ d^−1^) and 6 days (762.8 ± 29.7 µg C L^−1^ d^−1^) in the control and heated bay mesocosms, respectively (Fig. 2 and Supplementary Table S3). Separate analyses of data for each bay showed no statistically significant signature of a curvilinear (squared) relationship between bacterial production and incubation temperature in either bay mesocosms (Supplementary Table S1), arguing against the existence of a peak in performance at intermediate temperature followed by a decline at the higher temperatures tested in this study. Bacterial production in the heated bay mesocosms increased with increasing temperature at different rates depending on incubation time (effect interaction, ANOVA, *F*_2,63_ = 5.0, *P* = 0.0095) with the increase being steepest and with average production being highest after 6 days of incubation (Fig. 2 and Supplementary Table S1). The control bay mesocosms showed a somewhat different pattern with increasing bacterial production with elevated temperatures at different rates depending on incubation time (effect of interaction, *F*_2,62_ = 25.8, *P* < 0.0001; Supplementary Table S1) that continued to increase between 6 and 9 days of incubation (Fig. 2 and Supplementary Table S1).

Comparisons between bays showed higher overall production in the heated bay mesocosms than in the control bay mesocosms after 6 days, particularly at lower temperatures (<24 °C; main effect of bay, *F*_1,38_ = 8.3, *P* < 0.0001; 6–15 °C, 398.0 ± 199.1 µg C L^−1^ d^−1^ heated bay, 225.9 ± 62.9 µg C L^−1^ d^−1^ control bay) with both bays showing a similar increased bacterial production with raised temperature (Supplementary Fig. S3 and Supplementary Table S1). However, average bacterial production was significantly higher after 9 days of incubation in the control compared to the heated bay mesocosms (6–35 °C, 226.5 ± 138.1 µg C L^−1^ d^−1^ heated bay, 426.2 ± 110.4 µg C L^−1^ d^−1^ control bay), particularly at higher temperatures (effect of bay×temperature interaction, *F*_1,38_ = 9.6, *P* = 0.037), which in part reflected a larger drop in production between 6 and 9 days in the heated bay (Fig. 2, Supplementary Fig. S3, and Supplementary Table S1). With increasing temperatures, the control bay mesocosms showed higher production rates after 9 days of incubation between 16 and 35 °C (601.52 ± 145.62 µg C L^−1^ d^−1^) compared to the heated bay (275.52 ± 159.21 µg C L^−1^ d^−1^; effect of bay at day 9, *F*_1,38_ = 19.54, *P* < 0.0001).

### 16S rRNA gene amplicon diversity indices in response to temperature incubation

**Fig. 3:**
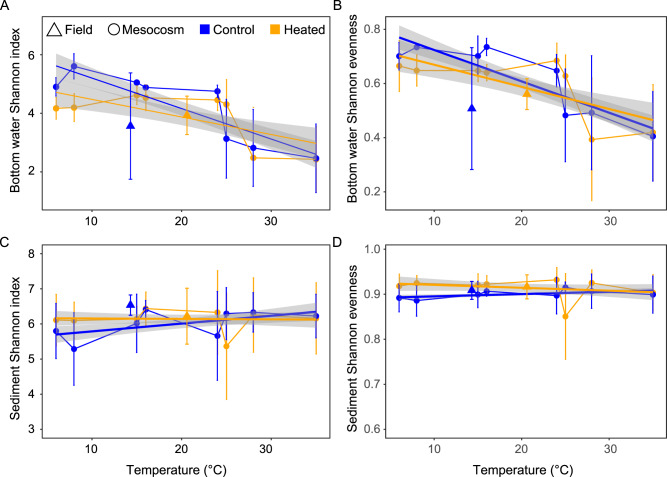
Bottom water and sediment microbial community alpha diversities from the field (coastal bays) and after 9 days of incubation at the different incubation temperatures (6–35 °C). Shannon´s H alpha-diversity index (**A**, **C**) values and the Shannon´s H evenness (**B**, **D**) for the bottom water (**A**, **B**; *n* = 9 field and *n* = 3 per incubated temperature) and surface sediment (**C**, **D**; *n* = 9 field and *n* = 3 per incubated temperature). The orange (heated bay) blue (control bay) points show the mean plus the standard deviation calculated from *n* = 9 and *n* = 3 replicates per bay, respectively. The blue and orange lines with gray shaded areas show the trend line added using the R geom_smooth function and the linear model (lm) method.

The sequenced temperature gradient incubation 16S rRNA gene amplicons from bottom water and sediment samples had on average 102,629 amplicon sequence variants (ASVs) per sample (min. 641, max. 659,354; Supplementary Tables S4 and S1). There was a tendency to a higher Shannon´s H bacterial diversity in the control bay bottom water incubations compared to the heated bay mesocosms (Fig. 3A) with significant decreasing values with higher temperatures in the control bay mesocosms (dropping from 5.2 ± 0.3 to 2.7 ± 0.9, *n* = 3 per bay) compared to a drop from 4.5 ± 0.3 to 2.7 ± 1.1 in the heated bay mesocosms (ANOVA, effect of temperature, *F*_9,56_ = 27.06, *P* < 0.001; Fig. 3A and Supplementary Table S1). A different pattern was observed for sediment Shannon´s H diversity (Fig. 3C) with a stable diversity with higher temperatures in both bays.

**Fig. 4:**
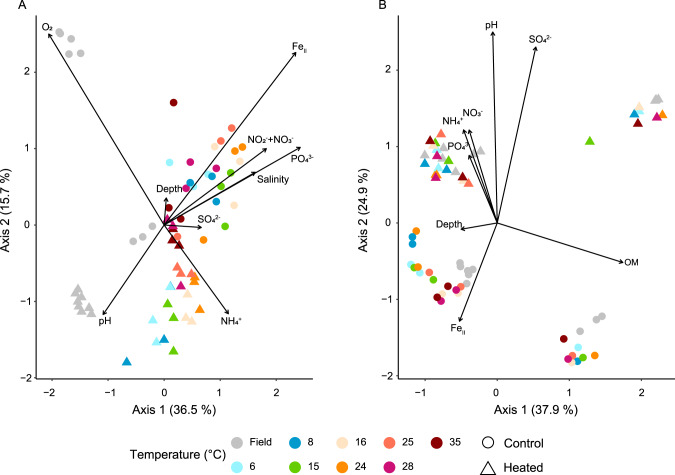
Distanced-based redundancy analysis (db-RDA) of the microbial communities. Shown are bottom water (**A**) and sediment (**B**) of the heated (triangle) and control (circle) bay for the field samples and the different incubation temperatures (6–35 °C). Plotted are the 16S rRNA gene samples based on relative abundance (using Bray–Curtis dissimilarities) of the sequenced ASVs in relation to the measured environmental variables (variance inflation factor (VIF) < 10).

The results of the distance-based redundancy analysis (db-RDA) showed an effect with incubation temperature for bottom water community relative abundances in the mesocosms from both bays (Fig. 4A). The main drivers of community-level change within the 2 bays field bottom water samples and mesocosms were oxygen (ANOVA, perm = 999, *F*_9,38_ = 2.7, *P* = 0.001), ammonium (*F*_9,38_ = 1.86, *P* = 0.015), and ferrous iron (*F*_9,38_ = 1.6, *P* = 0.041; Fig. 4A and Supplementary Table S1). The temperature gradient had a lesser influence on the surface sediment community composition with the samples mainly clustering with the bays on the first axis (42.4% variance explained) and sampling sites within each bay on the second axis (18.6%; Fig. 4B and Supplementary Fig. S4). The control bay sediment community showed a stronger composition change along the incubation temperature gradient with the main driver responsible for changes being organic matter (ANOVA, perm = 999, *F*_9,38_ = 4.7, *P* = 0.001), pH (*F*_9,38_ = 2.6, *P* = 0.013), sulfate (*F*_9,38_ = 2.5, *P* = 0.013), and ferrous iron (*F*_9,38_ = 2.1, *P* = 0.046; Fig. 4B and Supplementary Table S1).

### 16S rRNA gene amplicon community response to temperature incubation

The main phyla found in field sample bottom water of the two bays (corresponding to the microbial community at the start of the incubation; Supplementary Fig. S5) were Proteobacteria (43.5 ± 14.1 heated and 42.5 ± 17.5% control bay; average relative abundance of all samples in each bay), Cyanobacteria (13.4 ± 5.1 and 9.2 ± 4.7%), and Actinobacteria (10.4 ± 6.9 and 9.7 ± 3.2%). In addition, the highest relative abundance surface sediment phyla were Proteobacteria (30.2 ± 4.0 heated and 31.6 ± 4.4% control bay) followed by Bacteroidota (15.4 ± 1.9 and 20.4 ± 3.1%) and Desulfobacterota (10.5 ± 3.7 and 13.8 ± 1.9%).

**Fig. 5:**
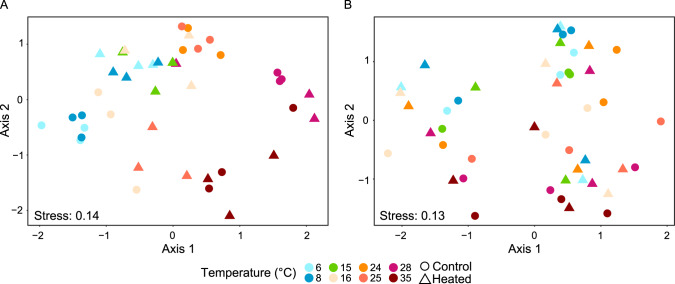
Non-metric multidimensional scaling (NMDS) based on Bray–Curtis dissimilarity of the response ASVs to the different temperatures within the gradient. Shown are the significant differential abundant ASVs (based Bray–Curtis dissimilarities of relative abundance of ASVs) that responded to the temperature changes in bottom water (**A**) and sediment (**B**).

Differential abundance analysis of significantly changed bottom water ASVs as a temperature response showed that lower temperatures (6–15 °C) selected for more similar bacterial communities in both bays, while higher temperatures (24–35 °C) selected for a different composition compared to the field conditions temperature (Fig. 5A). The higher temperature communities clustered in a similar direction on the first axis for both bays, while there was a difference between the bays on the second axis. A different temperature response was observed in the surface sediment communities with a strong influence of the sampling site on the first axis plus a similar influence at 25–35 °C on both bay communities on the second axis with higher temperatures drifting in a similar direction and lower temperatures (6–15 °C) clustered closer to their sampling location (Fig. 5B).

**Fig. 6:**
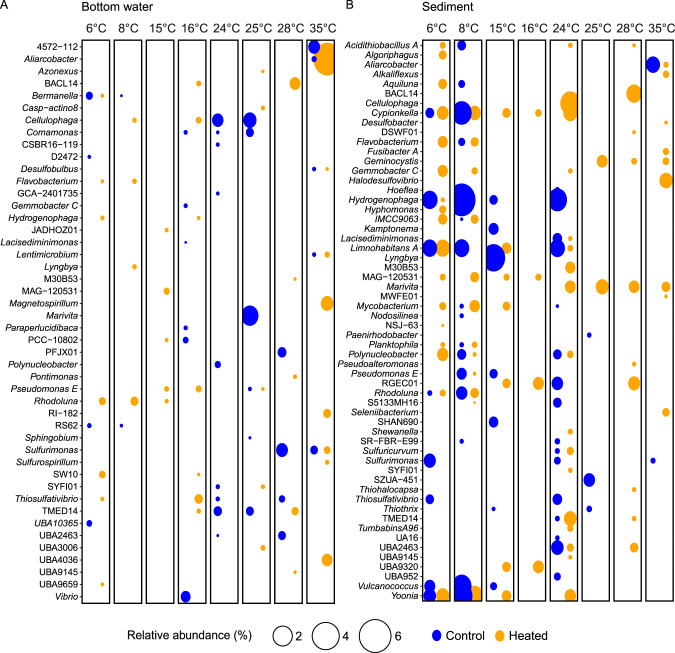
Balloon plot of significant differential abundant ASVs on genus level in bottom water and surface sediment. Shown are the ASVs on genus level that responded to changes in bottom water (**A**) and sediment (**B**) temperature. The *y* axis shows the different genera found after filtering taxa >1% of the relative abundance in each sample. The center of the circles indicates the related genus on the *y* axis.

An overview of the main phyla responding to temperature changes indicated that the temperature gradient had a strong influence on the bacterial communities of the bays (Supplementary Fig. S6). Proteobacteria had the strongest response showing significant changes in the relative abundance of ASVs (differential abundance analysis, Benjamin–Hochberg (BH) adjusted *P* < 0.05) followed by Bacteroidota and Cyanobacteriota in both bays mesocosms (Supplementary Fig. S6B). A closer look at significant differentially abundant ASVs at lower taxonomical ranks showed distinct relative abundance temperature responses between bottom water and sediment communities (Fig. 6). Significant changes within control bay mesocosms bottom water compared to 15 °C (field temperatures) showed increased taxa associated with the sulfur cycle at higher temperatures (28 and 35 °C) such as the sulfur-oxidizing genus *Sulfurimonas* (mean ± s.d. relative abundance for bay and temperature, 33.1 ± 1.8 (28 °C) and 6.6 ± 1.1% (35 °C)) that can use a variety of sulfur compounds as electron donor [32] and sulfate-reducing *Desulfobulbus* (0.6 ± 0.5% at 35 °C) that incompletely oxidize organic acids such as lactate, propionate, and ethanol to acetate coupled to dissimilatory sulfate reduction [33].

The control bay mesocosm sediments ASV response was especially observed at 24 °C with an increase of e.g., *Hydrogenophaga* (2.4 ± 1.3%), *Sulfuricurvum* (0.6%), and the sulfur oxidizer *Sulfurimonas* (0.7%) while at 35 °C a significant increase in the relative abundance of e.g., *Sulfurimonas* (0.6 ± 0.1%) and Aliarcobacter (previously known as Arcobacter [34]) (1.7 ± 3.3%) was observed (Fig. 6). The heated bay mesocosms sediment cores gave most significant changes compared to the field samples (*n* = 3, 20.6 ± 0.3) at 24 °C and fewest significant changes at 16 °C. For example, the strictly aerobic bacterium *Marivita* [35] relative abundance increased at warmer temperatures (mean ± s.d. relative abundance in the heated bay for the selected temperature range 1.1 ± 0.3%, 24–35 °C) while *Flavobacterium* (0.8 ± 0.08%, 6–8 °C) [36] and heterotrophic *Cypionkella* (1.5 ± 0.1%, 6–8 °C) [37] increased at lower temperatures. Finally, sulfate-reducing bacteria such as *Halodesulfovibrio* [38] relative abundance were 1.7 ± 1.4% at 35 °C.

### Distinct transcriptional response between heated and control bays

**Fig. 7:**
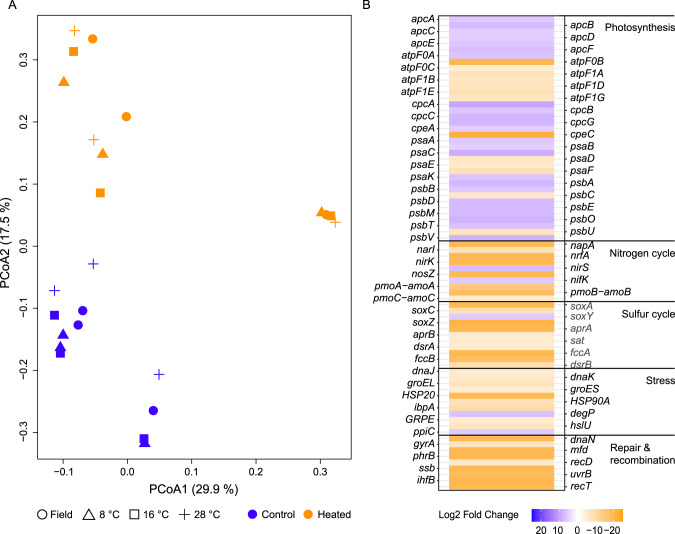
PCoA of metatranscriptomic reads plus differential expression analysis of selected genes between the sediment field samples. PCoA of the control (blue) and heated (orange) bay for the field and incubation temperature (8, 16, and 28 °C) metatranscriptomes (**A**). Significant differential RNA transcript numbers for open-reading frames with log_2_ fold change >5 (control vs. heated bay) of selected genes in the control (blue shades) and heated (orange shades) bays (**B**). High log_2_ fold change (blue) values indicate high transcript numbers in the control bay, while low (negative, orange) values indicate high transcript numbers in the heated bay.

The sediment sample transcripts showed strong clustering according to the sampling sites on the first axis (29.9%), while the two bays showed a clear separation on the second axis both in field conditions and after incubation (17.5%; Fig. 7A). The heated bay field and mesocosms did not show any clear temperature response while the control bay sediment warmer temperature incubations (28 °C) were more similar to the heated bay incubation samples showing a transformation towards the mRNA response to the temperature of the heated bay.

The sediment RNA transcripts were assigned to taxa (Supplementary Fig. S7) and then differential analysis comparing the field transcript profiles from the heated and control bays showed strong significant differences in energy metabolism, stress, and replication-associated genes (Fig. 7B). The control bay field samples (positive log_2_ fold change values) showed significantly higher differential transcripts for genes associated with photosynthesis such as photosystem I genes *psaABCK* (mean ± s.d. log_2_ fold change compared to heated bay; 5.61 ± 1.38). In contrast, the heated bay field samples showed significantly higher differential transcripts related to ATP generation (*atpF0BC* (−11.83 ± 6.05) and *atpF1ABDEG* (−8.55 ± 4.59)).

The dissimilatory nitrate reduction pathway (*nrfA* plus *napA* and *narI* also involved in denitrification, −18.4 ± 2.8), denitrification (*nirK* and *nosZ*, −18.8 ± 0.7), and nitrification (*pmoABC*-*amoABC*, −8.9 ± 4.6) genes had significantly higher transcripts in the heated bay field samples compared to the control bay. Genes assigned as involved in dissimilatory sulfate reduction (*aprAB*, *dsrAB*, and *sat*; −8.4 ± 4.9) had higher heated bay field transcripts suggesting increased sulfate reduction rates and the concomitant increased sulfur oxidation transcripts such as the SOX gene family (*soxACXYZ*, −18.3 ± 3.9) that converts thiosulfate to sulfate and sulfide oxidation *fccAB* transcripts (−18.5 ± 1.3). Transcripts assigned as related to heat stress, such as *HSP90A* (−7.2 ± 1.9), *HSP20* (−9.2 ± 5.7), *groES* (−9.9 ± 6.4), *groEL* (−7.7 ± 5.1), and *dnaJK* (−8.0 ± 5.4) were significantly higher in the heated bay field samples. Finally, significantly higher differential transcripts assigned to repair and growth were mainly found in the heated bay such as *dnaN* (−19.0 ± 0.001), *gyrA* (-9.7 ± 6.9), and *recT* (−19.1).

### Transcriptional response to temperature incubation

Differential analysis of all sediment RNA transcripts between field conditions and the incubation gradient showed a greater response across all incubation temperatures for the control bay samples while the heated bay showed a large RNA transcript response at the highest measured temperature (Supplementary Fig. S8).

**Fig. 8:**
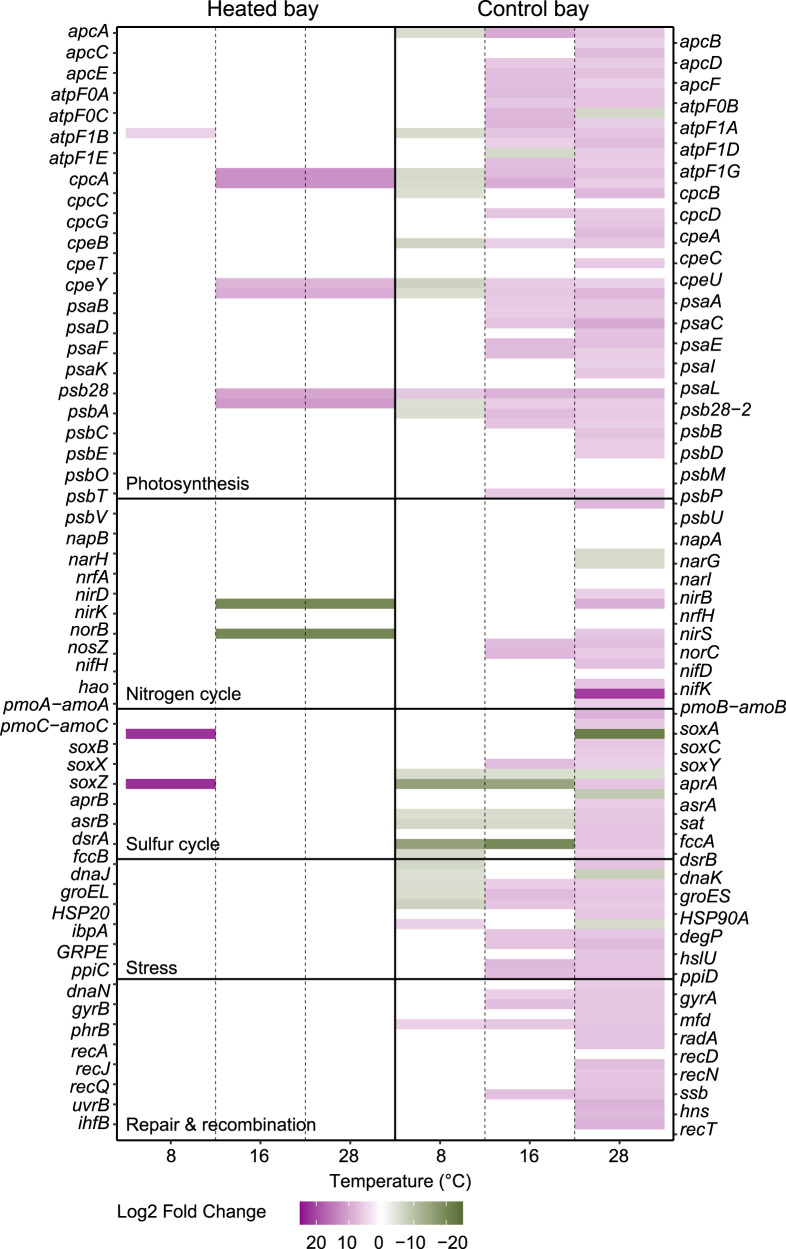
Differential RNA transcript numbers for selected genes from the control and heated bay sediment samples temperature response. Shown are the significant differential transcript numbers over log_2_ fold change >5 assigned to selected genes (field vs. experiment). Colors indicate if the gene had higher transcripts within the field (green shades) or at the indicated incubation temperatures (purple shades). High log_2_ fold change numbers (purple) indicate high transcripts in the incubation experiment samples compared to the field samples, while low log_2_ fold change (green) numbers indicate high transcripts in the field samples compared to the incubation experiment.

The control bay mesocosm sediment incubations had significantly lower transcripts at cold temperatures compared to the field samples. Higher transcripts were observed within higher temperatures (28 °C), which belonged to genes involved in energy metabolism, growth, and stress (Fig. 8). Most of the significant transcripts assigned to photosynthesis associated with the Cyanobacteria and increased at 16 °C and 28 °C compared to the field (12–15 °C). Transcripts encoding genes involved in the nitrogen cycle had significantly higher counts at 28 °C in the control bay mesocosms compared to the field. For example, these included nitrification or methane oxidation such as *pmoABC*-*amoABC* (mean ± s.d. log_2_ fold change compared to the field, 6.3 ± 3.8) assigned to e.g., *Methylobacter tundripaludum* and *Nitrosomonas* at 28 °C as well as transcripts involved in denitrification such as *nirKS* (3.8 ± 5.2) assigned to *Flavobacteriales* and *nosZ* (5.2 ± 0.7) assigned to *Alteromonadaceae* (Fig. 8 and Supplementary Fig. S9). Assimilatory and dissimilatory nitrate reduction genes were lower compared to the field in the control bay (e.g., *nirB* 1.3 ± 6.6) associated with *Syntrophobacter* and *nrfA* (3.67 ± 4.56) associated with e.g., *Ignavibacteriales* and *Deferribacteriales* (Fig. 8 and Supplementary Fig. S9). Control bay mesocosm incubation cores compared to the field samples had significantly lower transcripts related to dissimilatory sulfate reduction such as *aprAB* (−1.6 ± 6.2), *dsrAB* (−3.7 ± 3.1), and *sat* (−5.5 ± 0.2) at 8 °C and 16 °C and higher levels at 28 °C (1.6 ± 5.6; Fig. 8). Transcripts involved in the sulfur cycle assigned to taxa such as *Desulfovibrio* and *Desulfomaculum* were fewer at colder temperatures, while those assigned to genera such as the sulfate reducer *Desulfococcus* [39], the sulfur oxidizer *Beggiatoa* [40], and the purple sulfur bacteria *Thiocystis* [41] were higher at 28 °C compared to the field (12–15 °C) samples in the control bay (Supplementary Fig. S10). Genes assigned to the SOX complex, including *soxABXYZ* (5.90 ± 1.47) had significantly higher transcripts at 28 °C (compared to the field) and were assigned to *Thiocapsa*, *Thiobacillus*, and *Thiothrix* (Supplementary Fig. S10). Finally, transcripts assigned to stress-related genes such as *dnaJK*, *groELES*, *HSP20*, and *HSP90* were significantly fewer at colder temperatures (8 °C) while they were higher at 16 °C and were even more pronounced at 28 °C compared to the control bay field samples (Fig. 8). A similar pattern was observed for transcripts related to repair and recombination (e.g., *recAJNQ* and *gyrAB*).

In contrast to the control bay mesocosms, significantly differentially expressed heated bay mesocosm transcripts were mainly involved in photosynthesis and were higher at 16 °C and 28 °C such as PSI (mean ± s.d. log_2_ fold change compared to field samples ~21 °C, *psaAB*, 8.7 ± 0.5) and PSII (*psbBC*, 11.1 ± 0) associated with *Cyanobacteria* (Supplementary Table S3).

## Discussion

The IPCC states that continued climate change will result in more frequent marine heatwaves in ecosystems ranging from the poles to the equator [2, 42]. Most studies investigate either long-term warming to show potential gradual adaptation [43] or rapid short-term temperature increases to investigate the consequences of e.g., heatwaves [44]. In contrast, this study used amongst others metabarcoding and RNA-seq data of an incubation study from a heated bay (warmed for 50 years) in comparison to a control bay to examine whether and how long-term warming modulates the responses to short-term temperature shifts in coastal marine microbial communities.

The control and heated bay mesocosms reacted differently to temperature changes, with bottom water bacterial production in the heated bay having a broader thermal tolerance limit compared to the control bay. This provided evidence that long-term warming has influenced the community-level thermal sensitivity of microorganisms in the heated bay in ways that affect how they respond to short-term temperature changes (i.e., heatwaves). We cannot identify the mechanistic underpinnings based on the available data, but the flatter thermal performance curve of productivity in the heated bay (Fig. 2 and Supplementary Fig. S3) may reflect a difference in species composition and that the heated bay community might contain bacteria having broader thermal tolerances. The thermal performance curves were relatively linear in both bays, steeper in the control bay, and lacked a clear (statistically significant) peak at an intermediate temperature, suggesting the peak bottom water bacterial production concentrations were not reached within the used temperature range. This further suggested that, unlike what is typically reported within many species [29, 45], there was no clear trade-off between the peak production (height) and the temperature tolerance (breadth) of community-level bacterial production within the studied temperature range (6–35 °C). The differences over time and highest concentrations between the two bays may be related to the observed shift in the composition (Fig. 6) or relative abundance (Supplementary Fig. S10) of species in the community with thermal sensitivities that peak at different temperatures [27]. On a general level, the incubation study results supported previous observations that bacterial metabolic rates increase with higher temperatures [46–48]. However, the timing of the response differed between the bays. The bacterial production peaked earlier in the control bay compared to the heated bay mesocosms (after 6 days production was higher in the control bay mesocosm, while after 9 days it was higher in the heated bay). This temporal difference in the response may have been due to that bacterial production and abundance are not solely influenced by temperature [44] but rather, they depend on a combination of additional factors such as substrate availability [49, 50]. For example, the suggested increased activity in the heated bay microbial community [51] may have resulted in nutrient limitation in the closed incubation experiment and therefore, decreased bottom water bacterial production at higher temperatures from 6 days. However, no nutrient limitation could be observed in the measured environmental parameters after 9 days of incubation (Fig. 1). In contrast, bacterial production was still increasing in the control bay bottom water, suggesting nutrient limitation had not been reached. The bacterial production values found in the mesocosm bottom waters were higher than observed in other mesocosm studies even at the lower incubation temperatures [52–54]. The reason for this is unknown but may be related to the incubations including sediments, allowing for nutrient exchange between the sediment and the bottom waters. In general, the high bacterial production rates suggested future marine heatwaves might create short-term increased productivity at the base of coastal foodwebs.

Previous studies show that long-term exposure to elevated temperature reduces the diversity of bacterial communities [31, 55]. One reason could be the individual bacterial response to higher temperatures, while some bacterial taxa are sensitive others are resistant and therefore, can increase/decrease or be replaced [55, 56]. For example, the thermophilic taxa *Aquabacterium* and *Thiosulfativibrio* in bottom waters and *Desulfomicrobium* as well as *Mycobacterium* in sediment communities increased in relative abundances at 28 °C within the control bay. Other studies suggest that temperature alone does not have a significant influence on bacterial communities, whereas a combination of factors such as temperature along with nutrients [57] and/or acidification [58] causes a shift in community structure. The incubation experiment data confirmed a decrease in bottom water microbial diversity with the short-term temperature increase potentially affecting biotic (e.g., competition) and abiotic (e.g., nutrient) conditions.

The short-term incubations resulted in a rapid microbial transcriptional response to elevated temperatures such that the control bay sediment RNA transcript response was transformed to be more similar to the transcriptional profile of the microbial community observed in the sediment field samples in the heated bay. This was supported by the control bay incubation at 28 °C being closer to the heated bay field samples (Fig. 7) and by increased transcript numbers coding for e.g., energy metabolism such as dissimilatory nitrate reduction, dissimilatory sulfate reduction, and sulfur oxidation plus stress proteins as observed in the heated bay field data (Fig. 8). This was also underlined on the one hand by increasing relative abundances of nitrate-reducing bacteria such as Ignavibacteriales (*IGN3*), *Aliarcobacter*, and *Sulfurimonas* and on the other hand by decreasing porewater sulfate concentrations at higher incubation temperatures (Fig. 1) and elevated relative abundance of sulfate-reducing bacteria such as *Desulfatitalea*, *Desulfomicrobium*, *Desulfopila*, and *Desulforhopalus*. The transcriptional profile response of the control bay sediment samples becoming more similar to the heated bay may have been due to higher differential expressed transcripts suggesting elevated bacterial growth rates at increased temperature (+2 °C) [59]. The shift in control bay sediment transcripts towards the heated bay profile indicated a rapid transition of the bacterial metabolism, resulting in higher RNA transcripts assigned to energy metabolism with increased temperature that in turn, results in faster sulfate reduction closer to the sediment surface [51]. These results matched previous findings that investigated bacterial community composition, metabolic response, and sulfate fluxes between the heated and control bays showing increased microbial diversity in the surface sediment (0–1 cm). This was likely due to a shallowing of the geochemical zones due to elevated metabolic rates at increased temperatures consuming oxygen at the sediment surface that resulted in an increased diversity of taxa able to reduce a variety of anaerobic electron acceptors [51]. In addition, mRNA transcript sequencing identified higher differential transcript counts related to stress and repair, indicating the sediment microbial community’s temperature optima were below that of the water, resulting in a weakened resilience of the community [51]. This may result in toxic sulfide being generated closer to the sediment-water interface in addition to already expanding oxygen-depleted areas (as suggested by decreasing oxygen concentrations at higher incubation temperatures; Fig. 1). Furthermore, a marine heatwave event in 2018 demonstrated that coastal shores are highly responsive to ongoing changes in temperature, with elevated CH_4_ and CO_2_ concentrations measured afterward either due to increased microbial production or the heatwave related release of previously accumulated gases [8]. In summary, the observations suggested rapid transition to a state of elevated bacterial metabolic rates with resulting shallowing of the geochemical layers and an overall weakened resilience, which could potentially lead to fueling further effects of climate change resulting in a negative feedback loop.

“Tipping points” are often referred to as the reorganization of a system, often in a non-linear manner, which does not return to the initial state even after the drivers decrease again [4]. In human time scales, irreversible changes are already recognized for slow-to-respond processes such as deep ocean warming, acidification, and sea level rise in the ocean [42]. Furthermore, marine environment regional-scale tipping points such as heatwaves [60] or coastal hypoxia [61] have also been recognized. In contrast to the rapid transition of bacterial communities to warming conditions as discussed above, transcripts encoding e.g., energy metabolism, stress response, and repair mechanisms within the heated bay sediment samples under short-term incubation at lower temperatures did not become more similar to the control bay. This suggested either a tipping point had occurred and the metabolic response to temperature might not return to contemporary conditions or that it will take a longer time to do so as metabolic rates will be lower at reduced temperatures.

Higher temperatures lead to lower oxygen solubility in water [62] and reduced oxygen concentrations due to elevated microbial metabolic rates [46]. This results in decreased bottom water oxygen concentrations and suggested hypoxia/anoxia in surface sediments by decreased sulfate levels and the use of anaerobic electron acceptors, such as nitrate or sulfate for organic matter mineralization in sediments [62]. Both bays showed increased sulfate reduction and higher relative abundance of sulfate-reducing bacteria (e.g., *Desulfobulbus*) with elevated temperatures. The likely resulting increased sulfide concentrations led to higher relative abundances of sulfur oxidizers such as *Sulfurimonas*, *Sulfuricurvum*, and *Aliarcobacter* in bottom waters of incubations from both bays. These observations fit Broman et al. [63, 64] who showed that the relative abundance of *Sulfurimonas* and *Aliarcobacter* increase after oxygenation of anoxic sediments. In addition, the potentially increased sulfide from higher sulfate reduction rates within sediments may bind ferric iron and hinder the formation of phosphate-binding ferric oxyhydroxides [65]. Increasing Fe-P dissolution likely resulted in the observed higher phosphate concentrations in the pore waters [66] and their release into the water column. This has the implication of future heatwaves potentially triggering algal blooms in other ways than just warming [67].

## Conclusions

In the future, it is expected that marine heatwaves will influence bacterial communities with effects on nutrient- and energy cycling. Notably, short-term temperature fluctuations will become more common with future heatwaves and will likely lead to a rapid response in bacterial communities. This was experimentally supported by differences in geochemical parameters, a shift in both the bottom water and sediment 16S rRNA gene-based microbial communities at the various incubation temperatures, and the bottom water bacterial production response to increased temperature was larger in the control bay than in the heated bay mesocosms. In addition, the thermal tolerance curve was flatter and broader in the heated bay bottom water samples. This was also indicated in the surface sediment transcriptional profile of the mesocosms in which the control (i.e., contemporary climate) became more similar to the heated bay field conditions. Finally, there was no evidence for rapid return to the contemporary state of the long-term heated bacterial community species composition and metabolic response with lower temperatures within surface (0–1 cm) sediments. This also supported that a tipping point had occurred in the heated bay field sediment microbial community that was continually under heat stress.

## Materials and methods

Additional details of the procedures can be found within the supplemental methods. A previous study compared water column and surface sediments in a coastal Baltic Sea bay (57°25’09.7“N 16°40’19.3“E; Supplementary Table S4) that has been used for the past 50 years as a warm water discharge from a nuclear power plant to a non-impacted control bay (57°25’58.7“N 16°41’17.2“E; Supplementary Table S4) as a unique natural laboratory to investigate potential future climate change effects [51]. The location of the artificial heated bay is in southern Sweden, north of the city of Oskarshamn. The semi-enclosed bay is open in one direction to the Baltic Sea and has no known in-flow from any freshwater source. The thermal discharge resulted in an average water temperature increase of 5.1 °C (annual mean temperature in 2018, 18.29 ± 5.90 °C heated bay and 13.86 ± 7.25 °C control bay (Supplemental Fig. S11)) [31] that is in the predicted range of the RCP5-8.5 scenario (3.3–5.7 °C) by the year 2100 [42] compared to an unaffected control bay only connected by the open Baltic Sea with a distance of ~1.5 km [51]. The cooling water was at no time in direct contact with radioactive material and no significant influence of radiation occurred [51]. The control bay was chosen due to its close location (~1.5 km), without being affected by the warm water discharge. Although being located very closely to each other, both bays are natural systems that result in variations of environmental parameters, such as e.g., a thin freshwater plume observed in spring 2018 in the surface waters of the control bay [31]. However, previous studies of the two bays concluded that temperature was a key driver of changes in bacterial communities.

### Field sampling

Sampling was conducted at three sites in the two coastal Baltic Sea bays (heated bay and control bay; Supplementary Table S4) as described by Seidel et al. [51]. The field sampling (11 cores per sampling site, *n* = 66, Table 1) was conducted on 2 consecutive days (first the heated followed by the control bay) in May 2018 (mean (*n* = 3) temperature field heated bay 20.6 ± 0.26 °C and 14.3 ± 1.9 °C control bay) using acrylic cores (7-cm internal diameter, 60 cm length) and a Kajak gravity corer. Three cores per site (*n* = 9 per bay) were directly sacrificed in the field to collect bottom water and sediment samples for 16S rRNA gene amplicon sequencing (each *n* = 3, total *n* = 9 per bay), total RNA (each *n* = 1, total *n* = 3), and chemistry analyses (each *n* = 3, total *n* = 9). The remaining cores were transported to the laboratory and installed in the incubation experiment on the same day. The acrylic cores with sediment (mean ± s.d. height, 29.56 ± 4.47 cm) and bottom water (27.58 ± 4.82 cm) were incubated as a whole in the water bath.

**Table 1:** Overview of the sample numbers at various stages of the experiment and analysis.

Method	Type	Samples (*n*)	Information
Field sampling	Sediment cores	11	Per sampling site
		66	Total cores taken
		18	Zero time point
	BW (DNA)	66	16S rRNA gene amplicon sequencing
	SED (DNA)	66	16S rRNA gene amplicon sequencing
	SED (RNA)	24	Metatranscriptomics
	Chemistry	66	Various chemical parameter analysis
Temperature	Annual mean	6	3 × 2 bays water column temperature
Incubation mesocosm	6–35 °C	48	3 × 2 bays for 8 different incubation temperatures
Core height	SED	48	SED height measurements
	BW	48	BW height measurements
BP	BW	150	3 sites × 2 bays × 3 days × 8 temperatures × 6 zero time points
Alpha diversity	BW (ASVs)	66	
	SED (ASVs)	65	
Chemistry	Various BW or SED	66	If not otherwise stated, all samples were included in the analysis
	BW salinity	63	
	SED Fe_tot_	65	
Db-RDA	BW	65	
	SED	65	
nMDS	BW	66	
	SED	65	
Differential abundance analysis	BW	24	Within mesocosm comparison 3 × 2 per bay compared to 7 temperatures
		48	total
	SED	11	3 per sampling site compared to 8 temperatures (for each site)
		48	

*BW* bottom water, *SED* sediment.

### Incubation experiment set-up

Eight water baths containing 200 L water were placed in thermostat-controlled rooms and further heated/cooled (thermocontrol 150 W, EHEIM, Germany) to temperatures ranging from 6 to 35 °C (6, 8, 15, 16, 24, 25, 28, and 35 °C) as measured hourly via HOBOware sensors (Onset Computer Corporation, USA; Supplementary Table S4 and Table 1). Each water bath contained a frame (Supplementary Fig. S12) holding the cores in a stable position, so that the cores were heated/cooled along the whole sediment plus bottom water length. However, they were not fully submersed to ensure no contact between the bath water and water column inside the cores. Each core was closed with a lid, which had holes for oxygen circulation, and each core contained sterile tubes (Thermo Scientific) with neodymium magnets that were connected to the lid with fishing line such that they could freely rotate. The bottom water overlying the sediment cores was mixed inside the cores to prevent stratification and anoxia via magnets attached to a pump (Eurostar 20, IKA) set to rotate with a speed of 30 rpm in the middle of the tank (that the mixing maintained oxygenation of the water column was confirmed in previous studies [68, 69]). Lights (Osram Lumilux daylight, L 36 W/865) were installed above each tank and left illuminating the cores in a 16:8 h day–night cycle to imitate natural dim light conditions (6.1 ± 0.3 µmol/m^2^/s) [70].

### Sampling of the incubation experiment

Oxygen and pH were measured in situ on three and two occasions, respectively (Supplementary Table S4) close to the sediment surface, without disturbing it (Supplementary Fig. S1). Subsamples for bacterial heterotrophic production were collected from the field and on incubation days 3, 6, and at the end of the experiment (day 9) from the bottom water (Supplementary Table S3). Three technical replicates and one control with stopped bacterial activity (trichloric acid; 5% final concentration; Sigma-Aldrich) were taken from each experimental core. Bacterial production was estimated through the ^3^H-leucine incorporation method according to Smith and Azam [71] using commercial ^3^H-leucine (Perkin Elmer; 1 mCi ml^-1^) diluted to 1 μM with cold leucine [72]. Samples were incubated in the dark in the corresponding water baths for 1–2 h with a final concentration of 40 nM lukewarm ^3^H-leucine. Bacterial production rates were calculated with a cellular carbon-to-protein conversion factor of 0.86 kgC mol leu^−1^, an assumed proportion of leucine to total protein of 0.073%, and an isotopic dilution factor of 2, according to Simon and Azam [73].

After the incubation period, the cores were removed from the water baths and bottom water was collected and treated for 16S rRNA gene amplicon sequencing and chemical analysis as previously described in ref. [31]. The remaining water was decanted and the top (0–1 cm) sediment sliced and put into a 50 mL centrifuge tube as described by Seidel et al. [51]. The sediment was well mixed and directly 4.5 mL was transferred to a 15-mL centrifuge tube containing 0.5 mL of RNA fix solution, flash frozen, and stored at −80 °C until further analysis. The remaining sediment for 16S rRNA gene amplicon sequencing and chemistry analysis was sampled as previously described, cooled using ice packs while brought back to the laboratory the same day, and stored at −20 °C until further analysis to stop potential microbial processes according to Seidel et al. [51].

### Chemical measurements and DNA/RNA extraction

Chemical analyses were conducted on bottom water and porewater (centrifugation at 2200×*g* for 15 minutes) as well as sediment samples, according to Seidel et al. [31]. Homogenized bottom water and sediment DNA and sediment RNA were extracted and prepared and sent for sequencing to the Science for Life Laboratory (SciLifeLab), Stockholm. Extracted RNA was treated twice with DNase and sent for sequencing to the Joint Genome Institute (JGI) in Berkeley, USA.

### Sequencing, bioinformatics, and statistical analysis

16S rRNA gene amplicons were prepared and sequenced at the Science for Life Laboratory (SciLifeLab; Stockholm, Sweden, Supplementary Table S5). Taxonomy was assigned against the Genome Taxonomy Data Base (SBDI-GTDB, R07-RS207-1) set for DADA2, and the resulting data were analyzed using R [74] to give ASVs in bottom water and sediment (Supplementary Table S6). Further statistical analyses are described in the supplemental methods (Supplementary Table S1). Differential abundance analysis was performed on each dataset (bottom water and sediment) from the 16S rRNA gene ASVs (Supplementary Fig. S6 and Supplementary Table S7). For the metatranscriptome analyses, the pre-filtering of RNA data for quality control was performed by JGI and then the cleaned mRNA reads were co-assembled with Megahit [75] and gene calling was performed with Prodigal [76]. The predicted genes were assigned functions with eggNOG-mapper [77] against the eggNOG database [78] and taxonomy with EUKulele [79]. BBMap was used to map the reads from each sample back to the assembly and featureCounts [80] was used to summarize the counts for each gene. The count data (based on mRNA transcripts) together with functional and taxonomic annotations were further analyzed in R. The raw RNA-seq counts generated in the previous steps were filtered for at least five reads in at least three samples and then normalized using the median of ratios normalization method from the DESeq2 package (version 1.30.1). The normalized counts were used to construct a PCoA (Principal Coordinate Analysis) ordination based on Bray–Curtis dissimilarities (R package ecodist, version 2.0.7) [81]. Differential transcript analyses were based on raw counts using the DESeq2 package and significant (*P* adjusted <0.05) open-reading frames with a log_2_ fold change of at least five were kept and analyzed further (Supplementary Table S8).

## Supplementary information

Supplementary Information

Table S3

Table S4

Table S5

Table S7

Table S8

## Data Availability

The 16S rRNA gene sequencing data are available on the NCBI database under BioProject PRJNA739524 and PRJNA813295. RNA raw reads are available on the JGI Integrated Microbial Genomes and Microbiomes (IMG) database using the following references JGI proposal ID 503869.

## Code availability

The code to generate the figures, statistical testing, and Supplementary Table S6 can be found at https://github.com/laseab/CC_incub.
