# Supplementary material for: Climate change-related warming reduces thermal sensitivity and modifies metabolic activity of coastal benthic bacterial communities
Source: ISME J. 2023 Mar 28;17(6):855–69. doi: 10.1038/s41396-023-01395-z (PMC10202955; doi:10.1038/s41396-023-01395-z)
Supplement: Supplementary file 1 — Supplementary Information [file 41396_2023_1395_MOESM1_ESM.docx]

**Supplementary Information for**

Climate Change related warming reduces thermal sensitivity and modifies metabolic activity of coastal benthic bacterial communities

Laura Seidel^*^, Elias Broman, Emelie Nilsson, Magnus Ståhle, Marcelo Ketzer, Clara Pérez-Martínez, Stephanie Turner, Samuel Hylander, Jarone Pinhassi, Anders Forsman, and Mark Dopson

Laura Seidel

Email: laura.seidel@lnu.se

**This PDF file includes:**

Material and Methods

Figures S1 to S13

Supplementary Table Information S1 to S8

Table S1

Table S2

References (Material & Methods)

Material and Methods

Chemical measurements

Chemistry analysis was conducted on bottom water and sediment pore water (centrifugation at 2200 × g for 15 minutes) as well as sediment samples according to Seidel*, et al.* (1). Shortly, pH was measured for bottom and pore waters (pHenomenal, VWR pH electrode, VWR) and the iron and phosphate content were analyzed according to Seidel*, et al.* (2). Hach-Lange cuvette tests (LCK 353 Sulfate, LCK339 Nitrate, and LCK341 Nitrite) were used with a Hach-Lange spectrophotometer to analyze sulfate on bottom and pore water as well as nitrate and nitrite on pore water samples. Nitrate/nitrite concentrations in bottom water were analyzed using a modified nutrient analysis method for smaller volumes from Valderrama (3). Organic matter content (% wt) was estimated from sediment samples the via loss of ignition (LOI) method according to Seidel*, et al.* (2).

DNA and RNA extraction

Bottom water and sediment samples were prepared and DNA (0.25 g homogenized sediment) extracted (1), concentrations measured using Qubit 2.0 (Invitrogen, Life Technologies Corporation), and samples stored at -20°C until further processing. Homogenized samples for RNA extraction (~2 g sediment) were processed according to Seidel*, et al.* (2). The Illumina library for sediment and bottom water 16S rRNA gene DNA was prepared using the PCR primers 341f and 805r and a PCR program from Hugerth*, et al.* (4). Modifications of the process steps and Illumina adapters were done according to Lindh*, et al.* (5).

Extracted RNA was DNase treated twice with the Turbo DNA-free kit (Ambion) and concentrations were measured using Qubit (Invitrogen™) and NanoDrop (Thermo Scientific™) before they were sent to the DOE Joint Genome Institute (JGI) at the Lawrence Berkeley National Laboratory, Berkeley, USA. Illumina rRNA removal kits for Bacteria and Plant Leaf were used in a 50:50 ratio (Ribo-Zero, Illumina^®^). The TruSeq stranded Total RNA HT preparation kit was used according to the Illumina guidelines with 500 ng total RNA per sample starting material and eight cycles of PCR for the library preparation.

Sequencing, bioinformatics, and statistical analysis of the 16S rRNA gene amplicon data

16S rRNA gene amplicons were prepared and sequenced at the Science for Life laboratory (SciLifeLab; Stockholm, Sweden). The raw data (adapter removal and demultiplexing performed by sequencing facility) were filtered, trimmed, denoised, merged, and chimeras removed using the Nextflow (v. 22.04.4) built ampliseq (v. 2.4.0) pipeline (6) running on the UPPMAX cluster (Uppsala Multidisciplinary Center for Advanced Computational Science) with the following settings. Sequences were trimmed at 269 bp forward and 209 bp reverse and the – double primer settings were set to ‘true’ to run cutadapt (v. 3.4) twice to secure complete primer removal; the –sample_interference was set to ‘independent’ and after chimera removal, the taxonomy was assigned to the SBDI-GTDB (Sativa curated 16S GTDB database, FigShare. Doi: 10.17044/scilifelab.1486077.v4, R07-RS207-1). The average sequence count was 98,554 reads (min. 391 and max. 668,912; Table S6). The resulting data were analyzed using R version 3.5.2 (7) giving a total of 26,412 and 19,095 unique amplicon sequencing variants (ASVs) in bottom water and sediment (Table S6), respectively. As the sequenced data were compositional (8), the dataset was transformed, if not otherwise stated, to relative abundance (%) per sample for further analysis. Rarefaction curves (Figure S13) on raw reads were calculated in R (version 4.0.4) with the ‘vegan’ package version 2.5-6 (9). Cyanobacterial sequences were additionally compared against Phytoref (2017-04-04) and eukaryotic diatom sequences removed. For further analysis, samples with total <1,000 reads were removed (sample ‘X1006’, heated bay 25°C; Table S4). Calculations for alpha diversity were carried out using the SRS R package where normalizing was performed by scaling with ranked subsampling (10). To test if the alpha diversity differed between bays and varied along the temperature gradient, a general linear model was used to perform an ANOVA. The variables included ‘bay’, ‘sampling site nested in bay’ (three sites within each bay) as categorical variables, while ‘temperature’ (to test for linear relationship), and ‘temperature square’ (to test for curvilinear relationships) were used as continuous variables. Additionally, the two-way interaction of bay and temperature as well as bay and temperature square were included (‘lm’ function, ‘stats’ R package, version 4.0.4; Table S1).

A general linear model was used to perform an ANOVA to evaluate whether the bacterial production differed between the heated and control bay and varied according to incubation time and temperatures (Table S1). The initial model included the ‘bay’, ‘sampling site nested in bay’ (3 sites within each bay), and ‘time’ (3, 6, and 9 days) as categorical variables; ‘temperature’ (to test for linear relationships) and ‘temperature square’ (to test for curvilinear relationships) as continuous variables; and the effects of the two- and three-way interactions of the bay and time with temperature and temperature squared (Table S1). As the results showed significant effects for the two- and three-way interactions between bays, time, and temperature (Table S1); separate analysis of the associations with time and incubation temperature within each bay were performed to avoid over parametrization of the model (Table S1). Significant differences between time points within each bay were evaluated using the emmeans function from the ‘emmeans’ R package (version 1.5.4, (11); Table S1). Additionally, separate analysis to evaluate the effects of bay and incubation temperature at each of the three time points using ANOVA were performed (Table S1).

Data exploration for the measured environmental variables was done according to Zuur, Ieno and Elphick (12). Outliers were detected and removed for the environmental variables salinity (‘X1100’, ‘X1101’, and ‘X1102’, control bay field) and sediment total iron (‘X1008’, heated bay 35 °C; Table S4). A general linear model was used to evaluate potential differences between the heated and control bays and variation according to temperatures for the different environmental variables. The model included the ‘bay’, ‘sampling site nested in bay’ (three sites within each bay), ‘temperature’ (to test for linear relationships), and ‘temperature square’ (to test for curvilinear relationships) as continuous variables, and the effects of the two-way interactions of the bay with temperature and temperature squared (Table S1).

Potential influencing environmental variables on the microbial communities in both bays and temperatures were tested within a distance-based redundancy analysis (db-RDA). The samples ‘X1006’ (heated bay, 25 °C) and ‘X1111’ (heated bay, field) (Table S4) were excluded from the analysis due to missing geochemical variables and lack of knowledge of possible interactions between communities and environmental variables. The variance inflation factor (VIF) was used to investigate potential collinear environmental variables that would reduce the statistical power (Table S1). All tested environmental variables for the bottom water samples were retained while for the sediment samples the variable nitrite was excluded. The db-RDA (*n*= 65 bottom water, *n*=65 sediment) was based on the relative abundance of ASVs and environmental parameters using the `vegan` package in R. PERMANOVA (*n*=999 permutations, `stats` R package) was used to test for potential significant environmental parameters shaping the bacterial communities in bottom water and sediment. Permutations of the samples were performed within bays. Microbial community dissimilarities between the bays and incubation temperatures for bottom water (*n*=66) and sediment (*n*=65) were shown using a non-metric dimensional scaling based on Bray-Curtis dissimilarities (nMDS; Figure S4).

Bottom water and sediment ASVs on phylum level were visualized using the ‘ggplot’ function within the R package ‘tidyverse’ version 1.3.0 (13) (Figure S5). Differential abundance analysis was performed on each dataset (bottom water and sediment) from the 16S rRNA gene ASVs. In more detail, a zero-inflated negative binominal model was used to account for the large number of zeros within the dataset. For the sediment samples, each sampling site within the bays was analyzed separately due to the differences in microbial communities between the sampling locations (Figure S5). For this, the field samples of each sampling site (*n*=3) were compared to the temperature gradient (*n*=8). The differential abundance analysis was then conducted using the ‘DESeq2’ package in R (version 1.30.1) by importing the ASV taxonomy and counts (Table S8) (14). The model used within the analysis included the variable of the group field versus experiment and the different temperatures observed. Significant ASVs (Benjamin-Hochberg (BH) adjusted *p*<0.05) with a log_2_ fold change of five or higher were retained and named in the following as ‘response ASVs’, showing the response of different temperature on the natural communities after nine days of incubation. However, a potential bias due to the incubation (bottleneck effect) could not be ruled out with this method. For the bottom water samples, a different approach was used to minimize the effect of the incubation study on the results, as the sampling sites were less diverse compared to the sediment samples. The analysis was carried out on each bay (heated and control) separately, but all sites within the bay (*n*=3 per bay) were used as replicates. Therefore, the temperature within the incubation study closest to the field *in situ* bottom water temperature (i.e., heated bay 24 °C and control bay 15 °C) was compared against the other incubation temperatures to investigate potential temperature related responses by the microbial communities. The model used to carry out the analysis used the observed temperature as a fixed variable and the contrast function to compare the temperatures with each other. The significant (*p*<0.05) ASVs with a log_2_ fold change of five or higher were used as response ASVs.

Sequencing and bioinformatics analysis of metatranscriptome data

Pre-filtering of RNA data for quality control was performed by JGI. In more detail, contaminants and Illumina adapters from the raw reads were removed and quality trimming was conducted with BBDuk version 38.51 (settings: rna=t trimfragadapter=t qtrim=r trimq=0 maxns=1 maq=10 minlen=51 mlf=0.33 phix=t removeribo=t removehuman=t removedog=t removecat=t removemouse=t khist=t removemicrobes=t mtst=t sketch kapa=t outribo=12965.2.300794.TTCAGGAG-CTCCTGAA.rRNA.fastq.gz clumpify=t tmpdir=null barcodefilter=f trimpolyg=5) (15). BBMap was used to map and remove reads that had at least 93 % identity with human, cat, dog, and mouse reference genomes as well as reads that mapped to common microbial contaminants, known-spike-ins, and ribosomal RNA, supplied with the software. The JGI cleaned mRNA reads were then co-assembled with Megahit (version 1.2.9) with default settings (16) followed by gene calling with Prodigal (version 2.6.3; settings: -p meta) (17). The predicted genes were assigned functions with eggNOG-mapper (version 2.0.8-2) with default settings (18) against the version 5.0 eggNOG database (19) and taxonomy with EUKulele (version 1.0.4, settings: -d phylodb -m mets) (20). BBMap (version 37.62, default settings) was used to map the reads from each sample back to the assembly and version 2.0.1 of featureCounts (settings: -t CDS –g ID) (21) was used to summarize the counts for each gene. The count data (based on mRNA transcripts) together with functional and taxonomic annotations were further analyzed in R.

Statistical analysis of the metatranscriptomic data

The raw RNA-seq counts generated in the previous steps were filtered for at least five reads in at least three samples and then normalized using the median of ratios normalization method from the DESeq2 package (version 1.30.1). The normalized counts were used to construct a PCoA (Principal Coordinate Analysis) ordination based on Bray-Curtis dissimilarities (R package ‘ecodist’, version 2.0.7) (22). Differential transcript analyses were based on raw counts (low counts filtered) using the ‘DESeq2’ package. For comparing both bays at the field conditions for the differential expression analysis, a model was used with bay and temperature as fixed variables. The contrast function was then used to compare the heated and control bay field samples with each other. Significant (*p*-adjusted <0.05) open reading frames (ORFs) with a log_2_ fold change of at least five were retained and analyzed further (Table S3). Potential changes in ORF transcript abundance as a response to the different incubation temperatures were tested on each sampling site within each bay. The model included groups (field vs. experiment) and temperatures (field, 8, 16, and 28 °C) as fixed variables. The contrast function was selected for comparing field against each incubation temperature for each sampling site (three per bay). Significant (*p*-adjusted <0.05) log_2_ fold change of at least five and higher were kept and further analyzed. Heat maps to show potentially differential expressed genes and bar plots to investigate potential organisms expressing these genes were generated using ‘ggplot2’ (version 3.3.5) in R (23).

Supplementary Figures

**
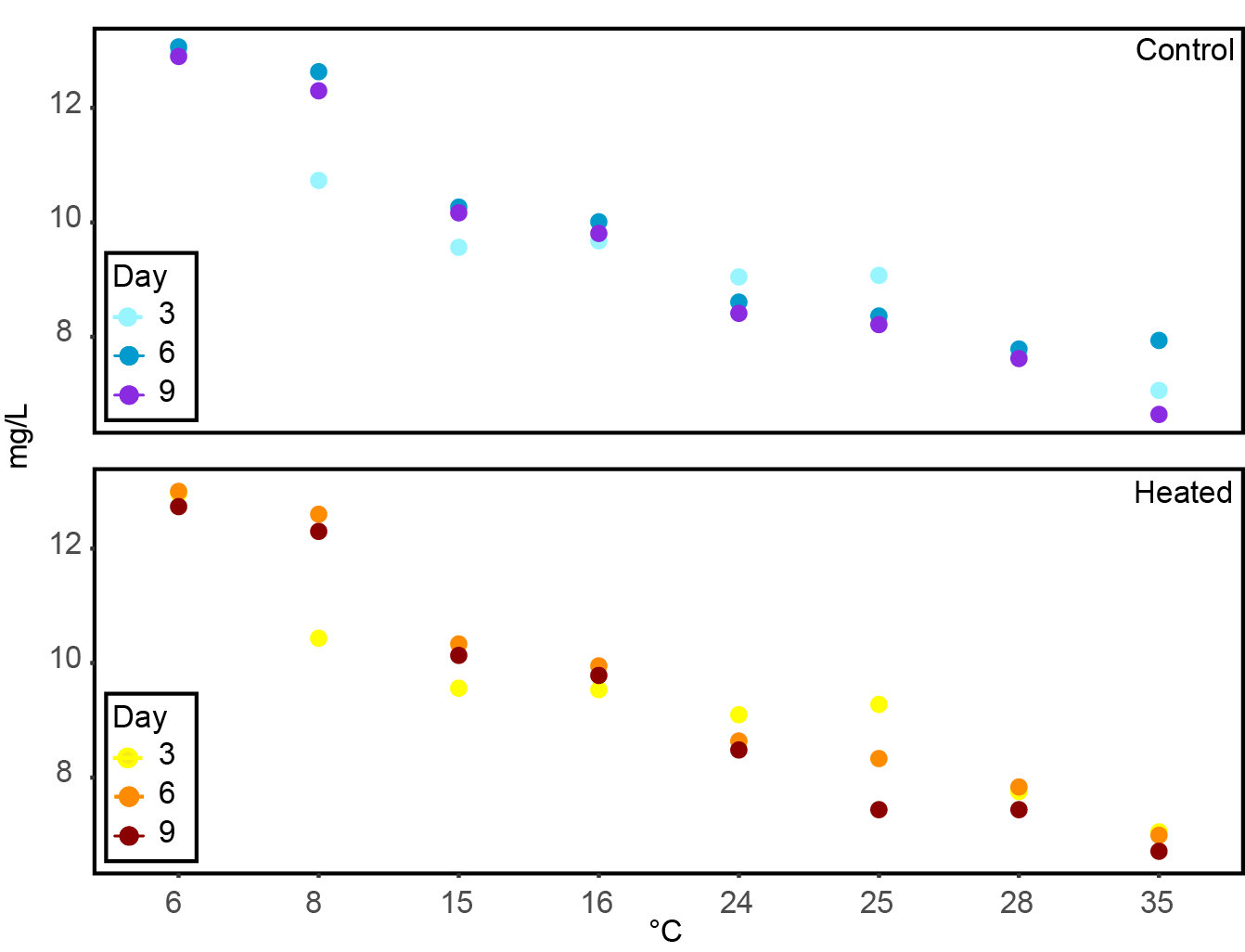
**

**Fig. S1. Oxygen tracking during the incubation.** The figure shows the measured oxygen concentration (mg/L, *n*=3) after 3, 6, and 9 days of incubation for the heated (lower half) and control bay (upper half) at the different temperatures of the gradient (6-35 °C).

**
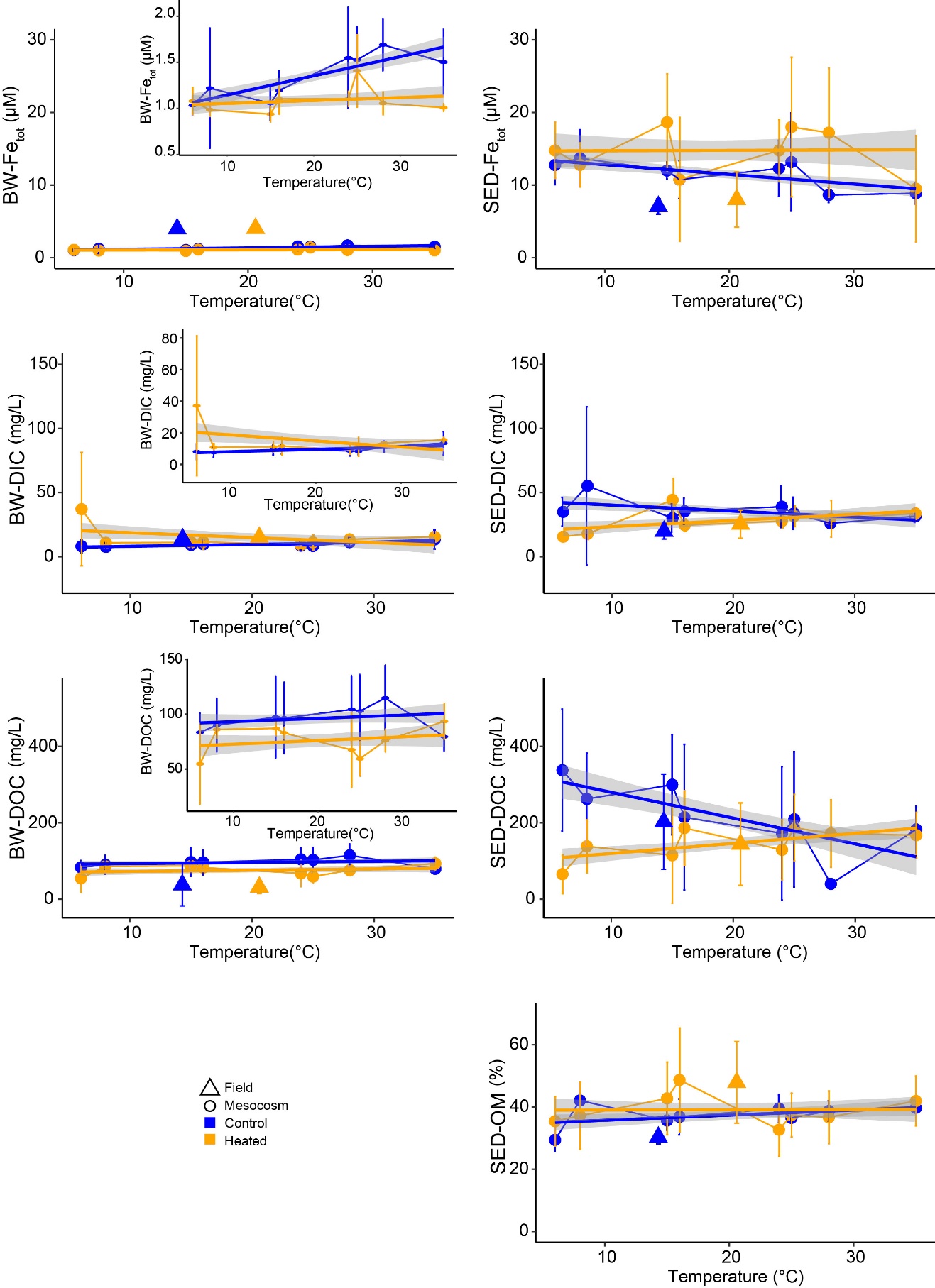
**

**Fig. S2. Environmental variables measured in the field and during the incubation.** The different geochemical parameters were measured in the bottom water and pore waters (0-1 cm sediment) of the collected field and incubation samples in the heated (orange) and control (blue) bays. A total of *n*=9 samples per bay for bottom water (*n*=18) and pore water (*n*=18) were collected for the field (triangle), while *n*=3 samples per bay per temperature for bottom water (*n*=48) and pore water (*n*=48) were collected for the mesocosm (circle) samples. In detail, data for total iron, organic matter, dissolved inorganic carbon (<0.7 µm), and dissolved organic carbon (<0.7 µm) were analyzed. Each point shows the mean *n*=9 or *n*=3 cores per bay (for field or per temperature), respectively plus the error bar shows the standard deviation.

**
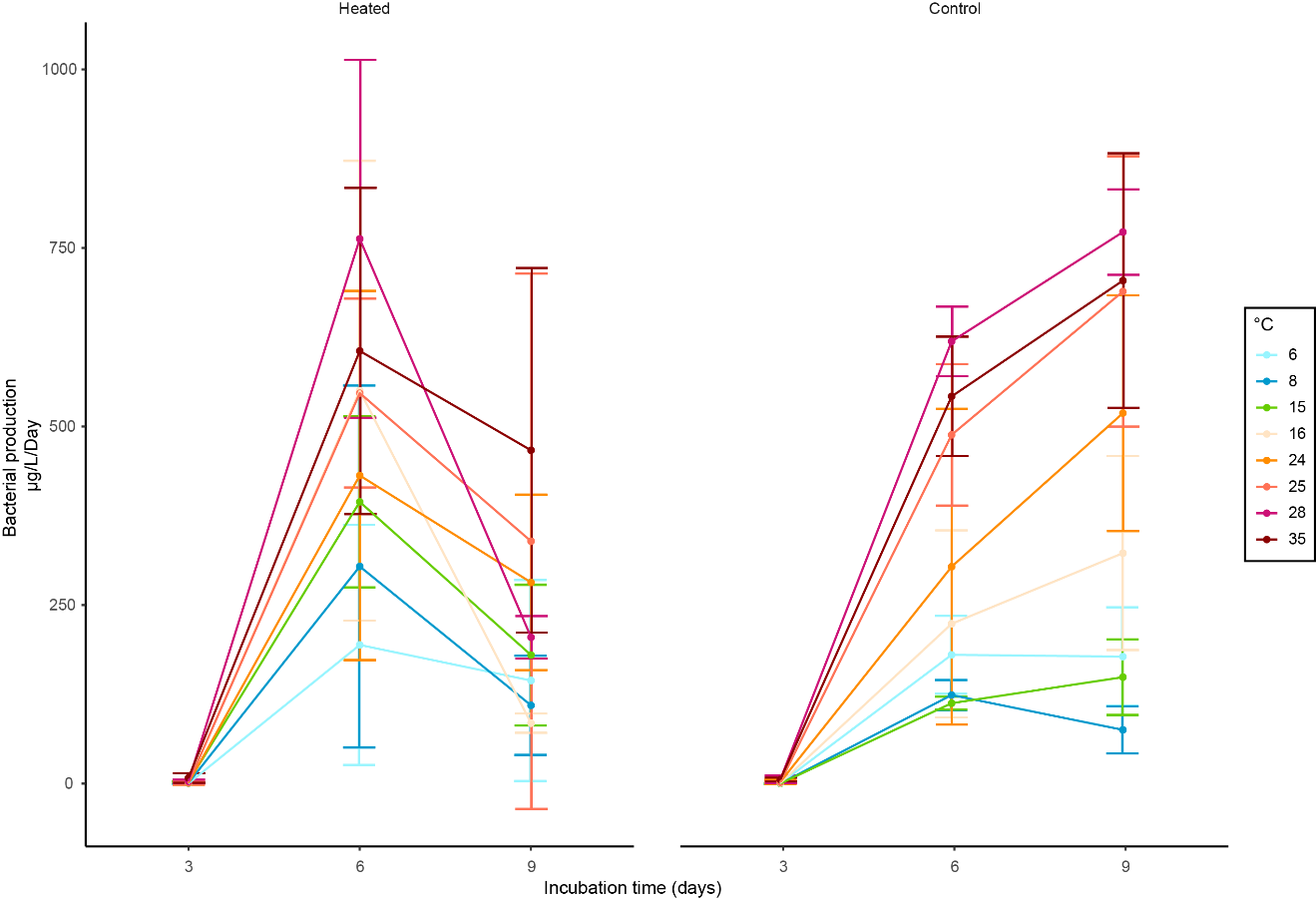
**

**Fig. S3. Bacterial production of the heated and control bay at the different incubation days.** Shown are the bacterial production concentrations in µg/L/d (linear scale) for the heated (left) and control (right) bay at three, six, and nine days (x-axis) of incubation at different temperatures (6-35°C). Each circle shows the mean (*n*=3) of the bays with standard deviation shown as error bars.

**
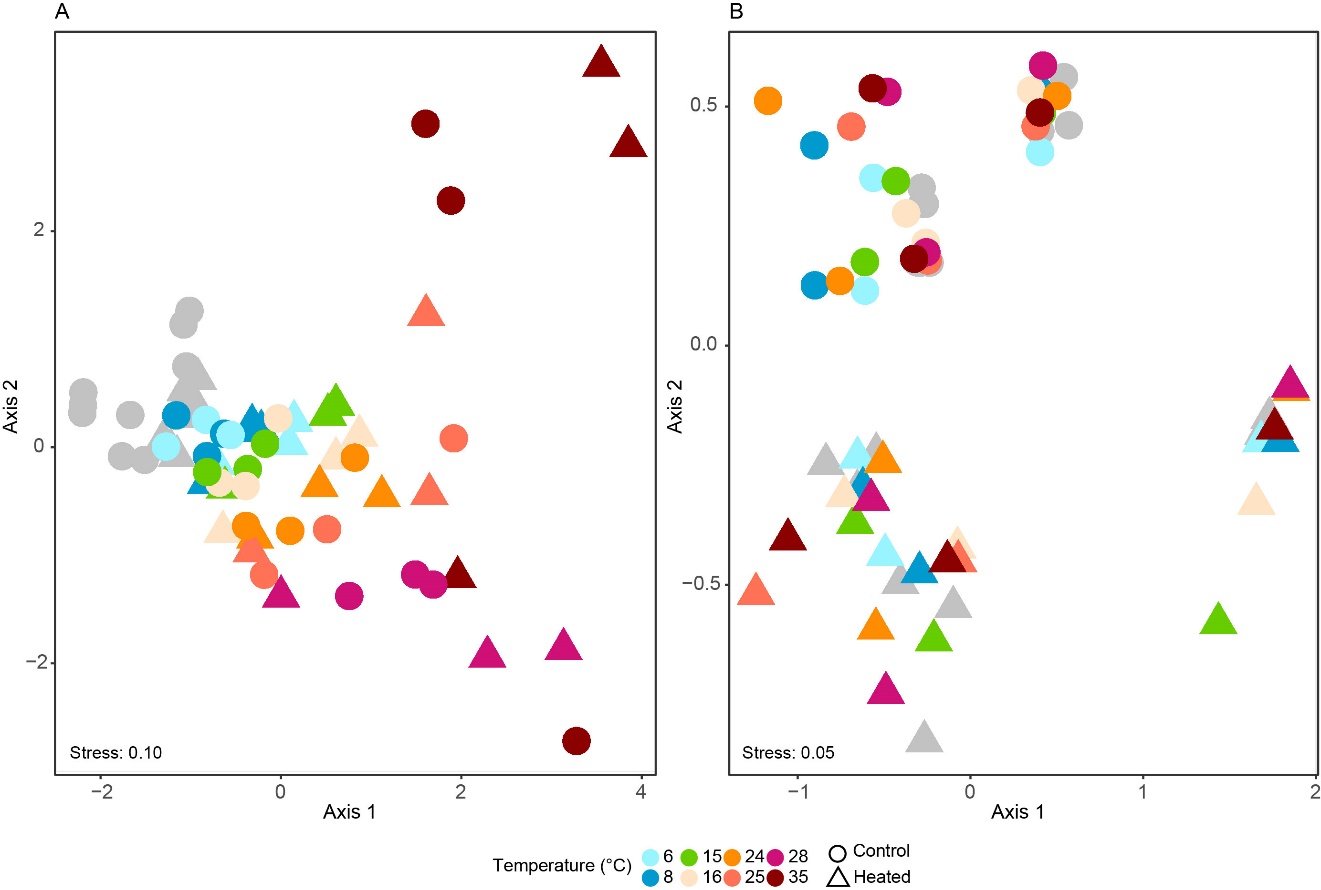
**

**Fig. S4. Non-metric multidimensional scaling of the collected samples.** Shown are the distances based on Bray-Curtis dissimilarities between collected bottom water (left) samples and sediment (right) samples in a two dimensional plot. The control bay samples are indicated with circles while the heated bay samples are indicated with triangles. The colors show the different collected samples from the field (grey) and the different temperatures from 6-35 °C (color gradient light blue to dark red); the stress values are shown in the left corner of each plot.

**
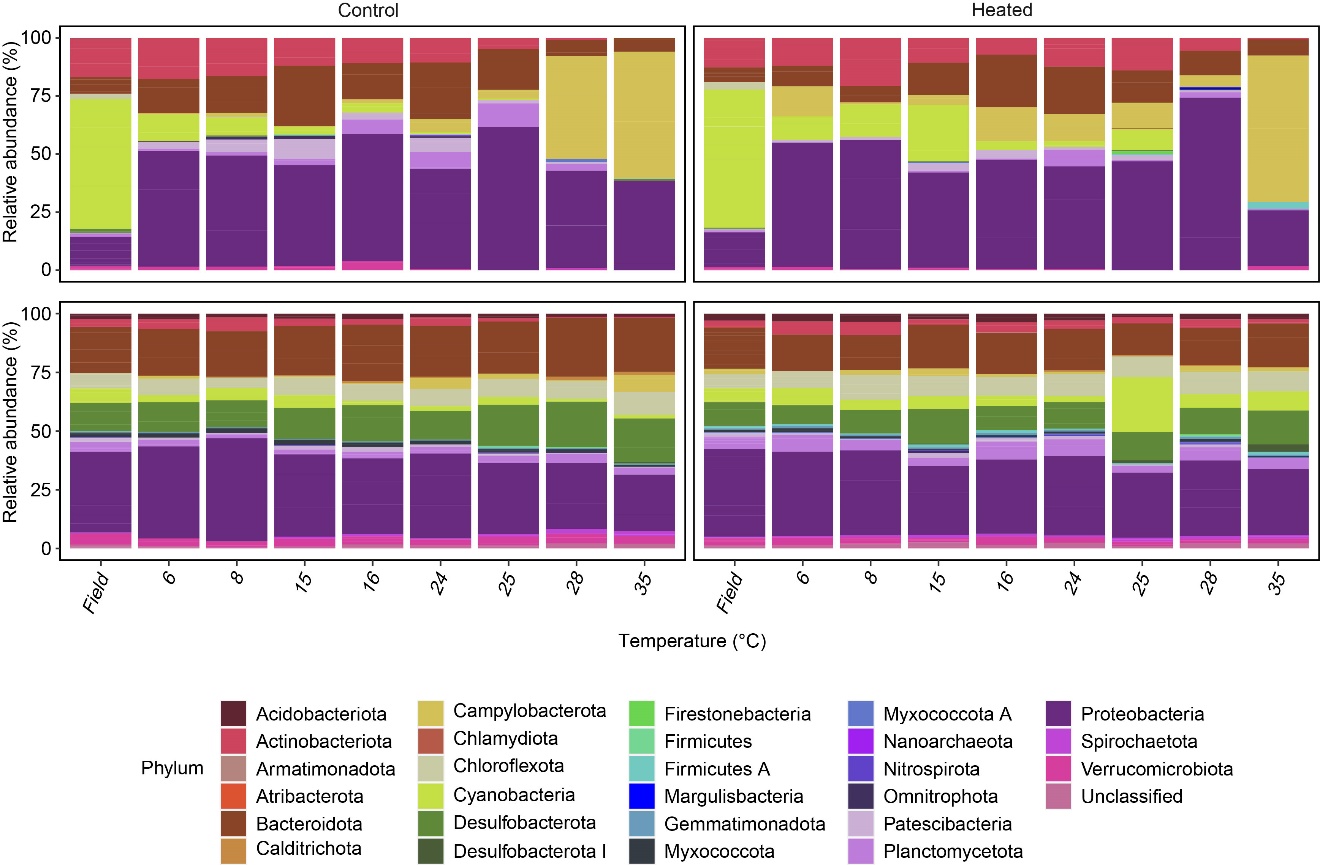
**

**Fig. S5. 16S rRNA gena ASVs on phylum level of the field and incubation samples.** Overview of the ASVs annotated on phylum level for the control (left) and heated (right) bays within bottom water (top) and sediment (bottom) samples. On the y-axis are shown the relative abundance scaled to 100 %, with each temperature measured samples (6-35 °C) and field samples on the x-axis. Each bar plot consists of *n*=3 samples; shown are the relative abundances of each phylum per samples above 0.5 %.

**
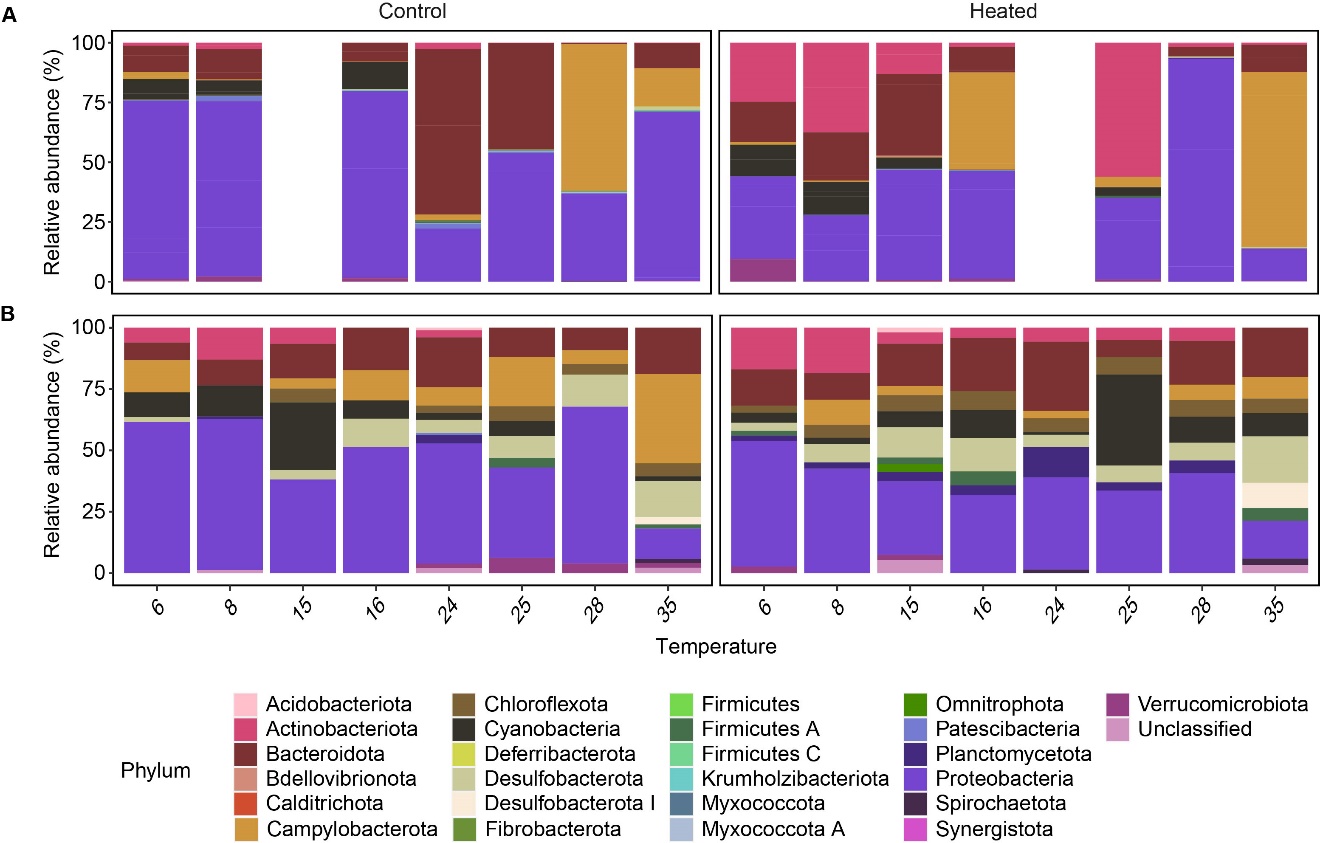
**

Fig. S6. Temperature response ASVs on phylum level in bottom water and surface sediment. Overview of the response ASVs annotated on phylum level from the differential abundance analysis on bottom water (A) and sediment (B) samples within the control bay (left) and heated bay (right). The y-axis shows the relative abundance scaled to 100 %, while the x-axis shows the samples for the different temperatures measured (*n*=3 per column). Differential abundance analysis was compared in bottom water to the temperatures within the incubation closest to the field conditions, when the samples were taken, to reduce potential effects of the incubation itself (missing 15 °C for control and 24 °C for the heated bay). For the sediment each sampling site had to be analyzed individually due to their high variation, therefore field samples were compared to each temperature for the sampling sites.


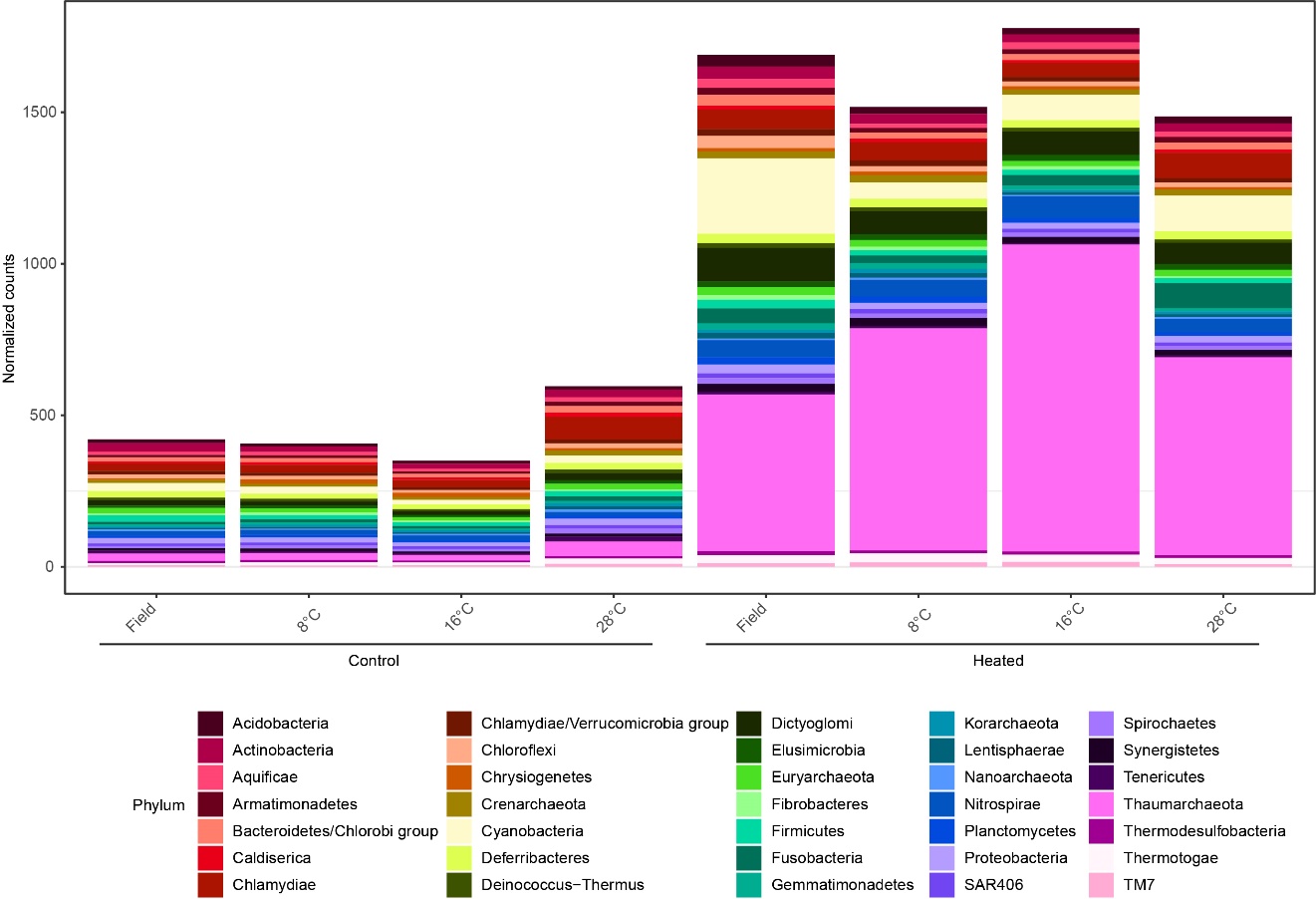


Fig. S7. Overview of annotated prokaryotes (Bacteria & Archaea) on phylum level of the sequenced RNA. Shown are the transcripts (ORFs) of the sequenced field, 8, 16, and 28 °C samples for the control (left) and heated (right) bay. The y-axis shows the normalized counts (DESeq2 median of ratios normalization, for between samples and DE analysis).


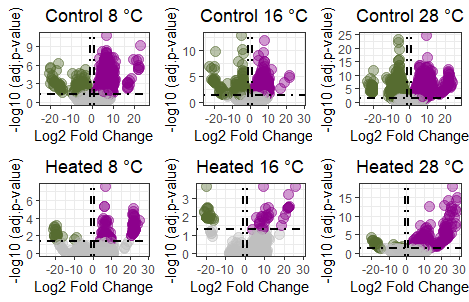


Fig. S8. Overview of differential expressed transcripts comparing field condition with incubated temperatures. Shown are the log2 fold change on the x-axis and the –log10 adjusted *p* value on the y-axis. The upper three graphs show the control bay field samples compared to 8, 16, and 28 °C incubation temperatures, while the bottom graphs show the heated bay field samples compared to the incubation temperatures; green indicate significant increase transcripts in the field samples; magenta shows significant increase in the incubation samples; grey states the non-significant (stable) transcripts; the black dotted line indicates the threshold for log2 fold change (diagonal) above 1 and non-significant transcripts (horizontal).


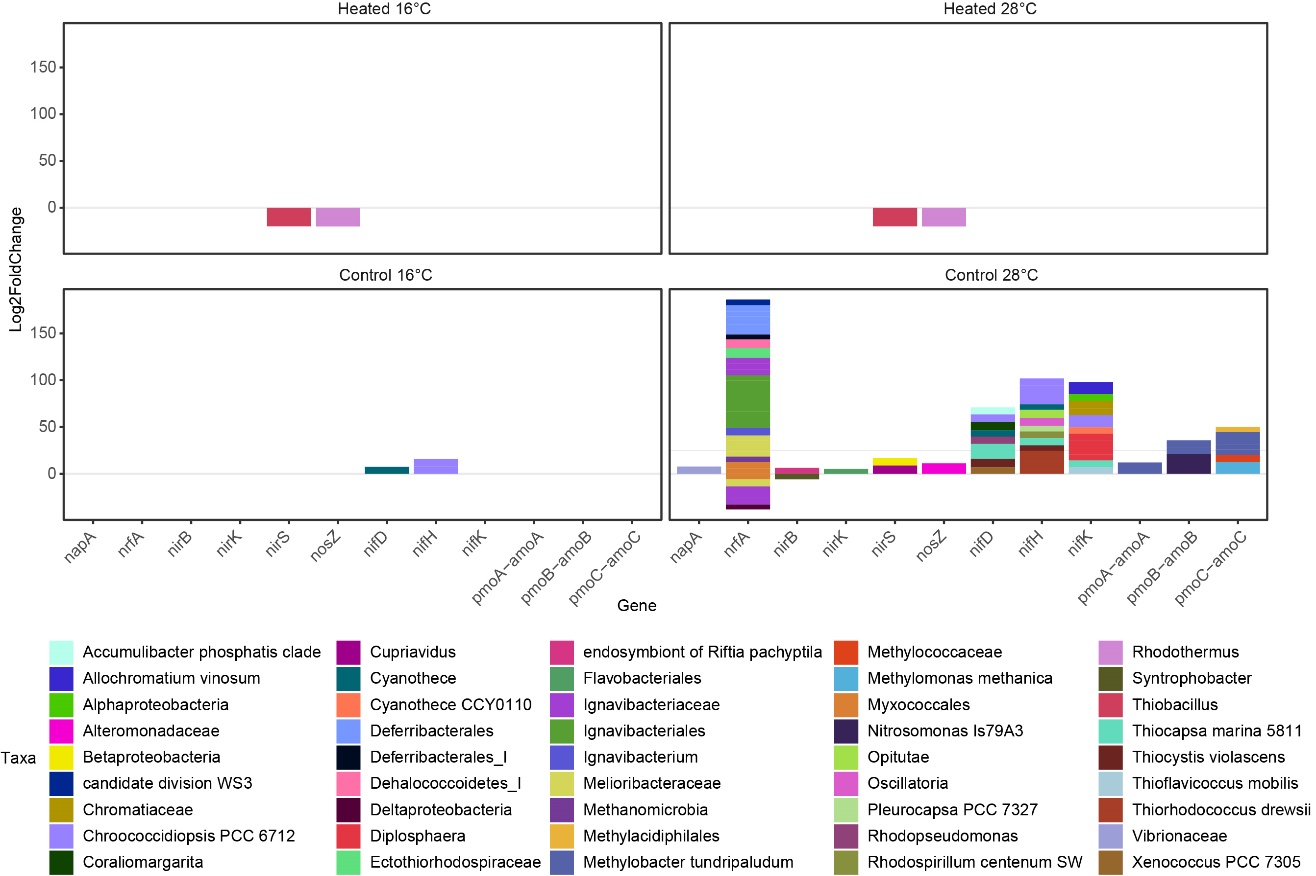


Fig. S9. Associated taxa of the genes related to nitrogen cycle in response to temperature changes of the control and heated bay. Overview of selected genes associated with taxa on lowest associated level of the differential expression analysis. Shown are only transcripts with a log fold change of >5 incubation samples (or <-5 field samples).


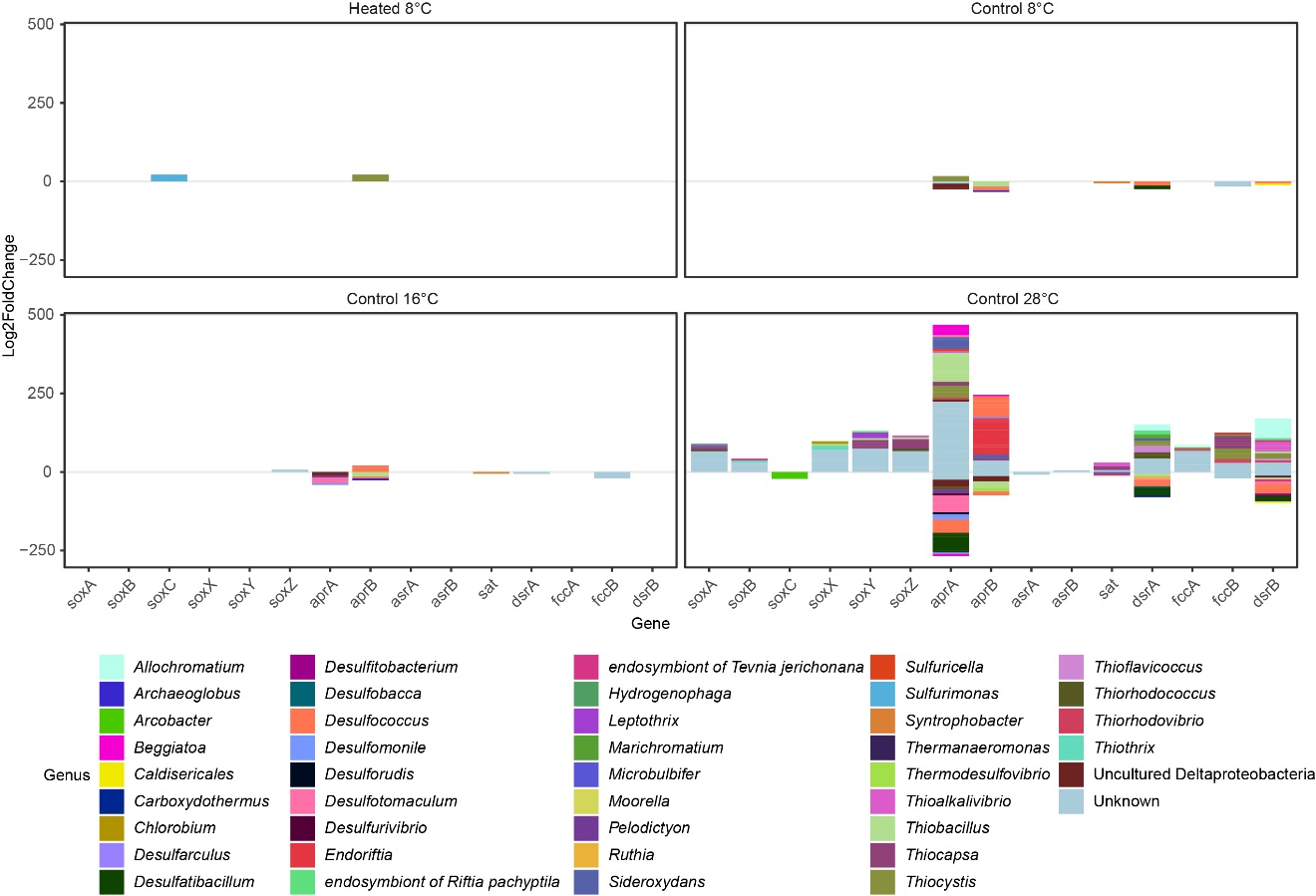


Fig. S10. Associated taxa of the genes related to the sulfur cycle in response to temperature changes of the control and heated bay. Overview of selected genes associated with taxa on genus level of the differential expression analysis. Shown are only transcripts with a log fold change of >5 incubation samples (or <-5 field samples).


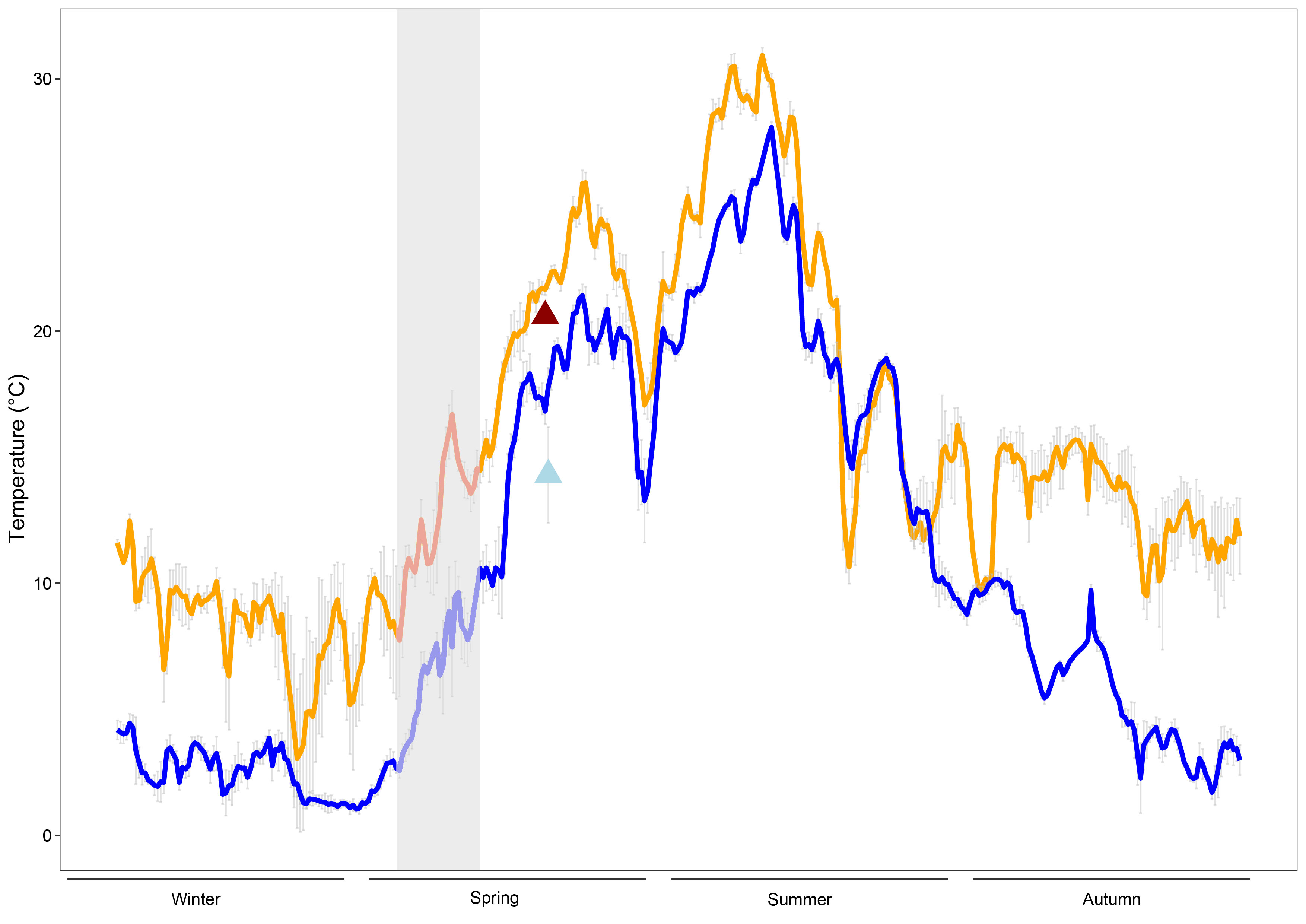


**Fig. S11. Temperature tracking 2018**. Overview of the daily average temperature fluctuations in the heated (orange; *n*=3) and control (blue; *n*=3) bay from January (left) to end of December (right) 2018 with standard deviations shown in light grey. The dark red triangle shows the average temperature from the three sampling sites samples taken (May 2018) in the heated bay while the light blue shows the average of the control bay sites. The light grey shaded area shows the temperature fluctuations of the month prior to sampling.


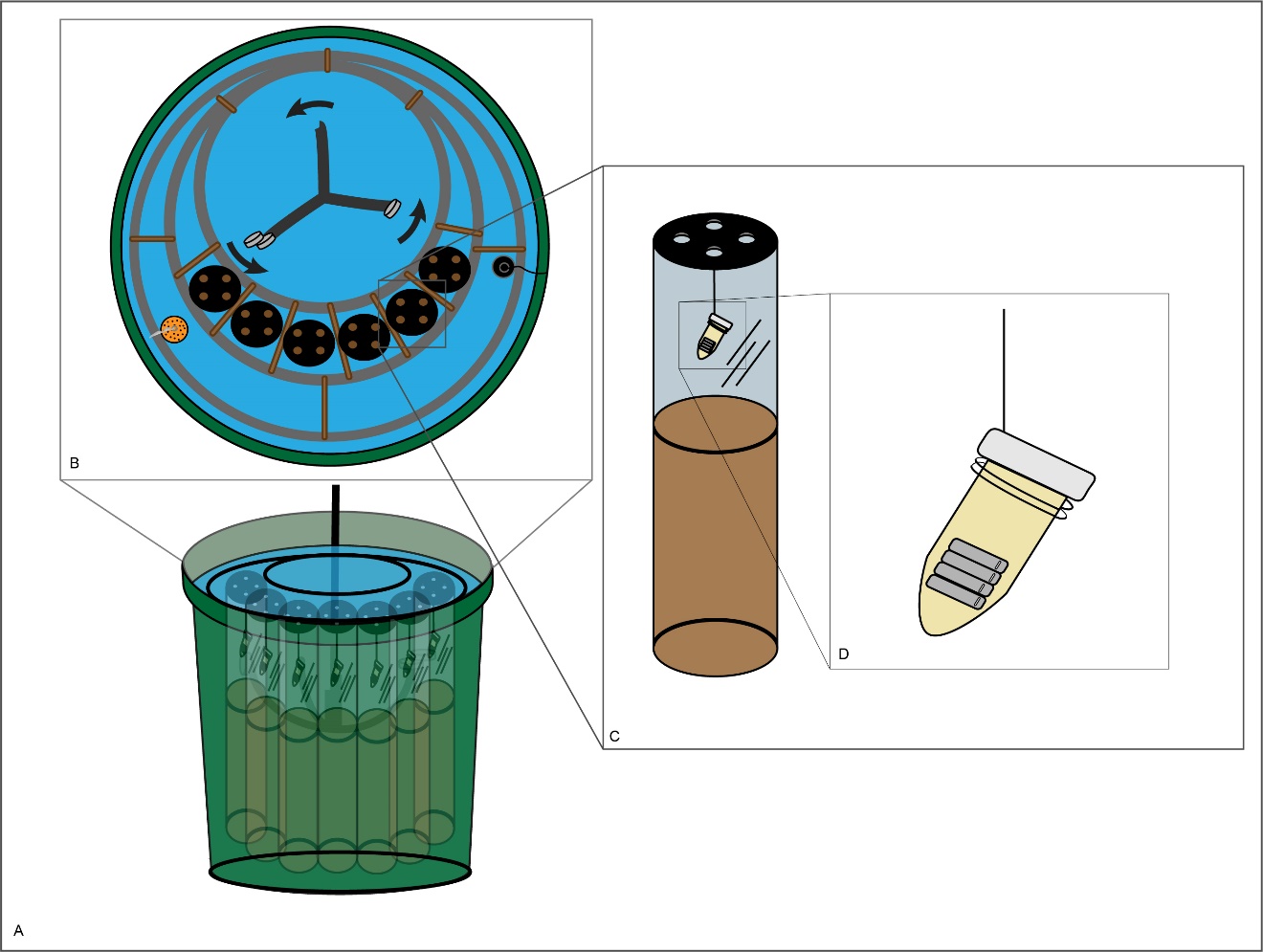


Fig. S12. Experimental Set-up. Schematic set-up of the experimental water bath with (A+B) position of the taken cores within the tanks and the customized frame; C shows the set-up of the individual cores with an open lid for air circulation and the attached tube (D); D shows the set-up of a tube on a fishing cord within each core, containing magnets, to enable free rotation within the cores. The hook in the middle of each tank (A), containing magnets, rotates and keeps the small tubes moving to allow water circulation during the whole experiment.

**
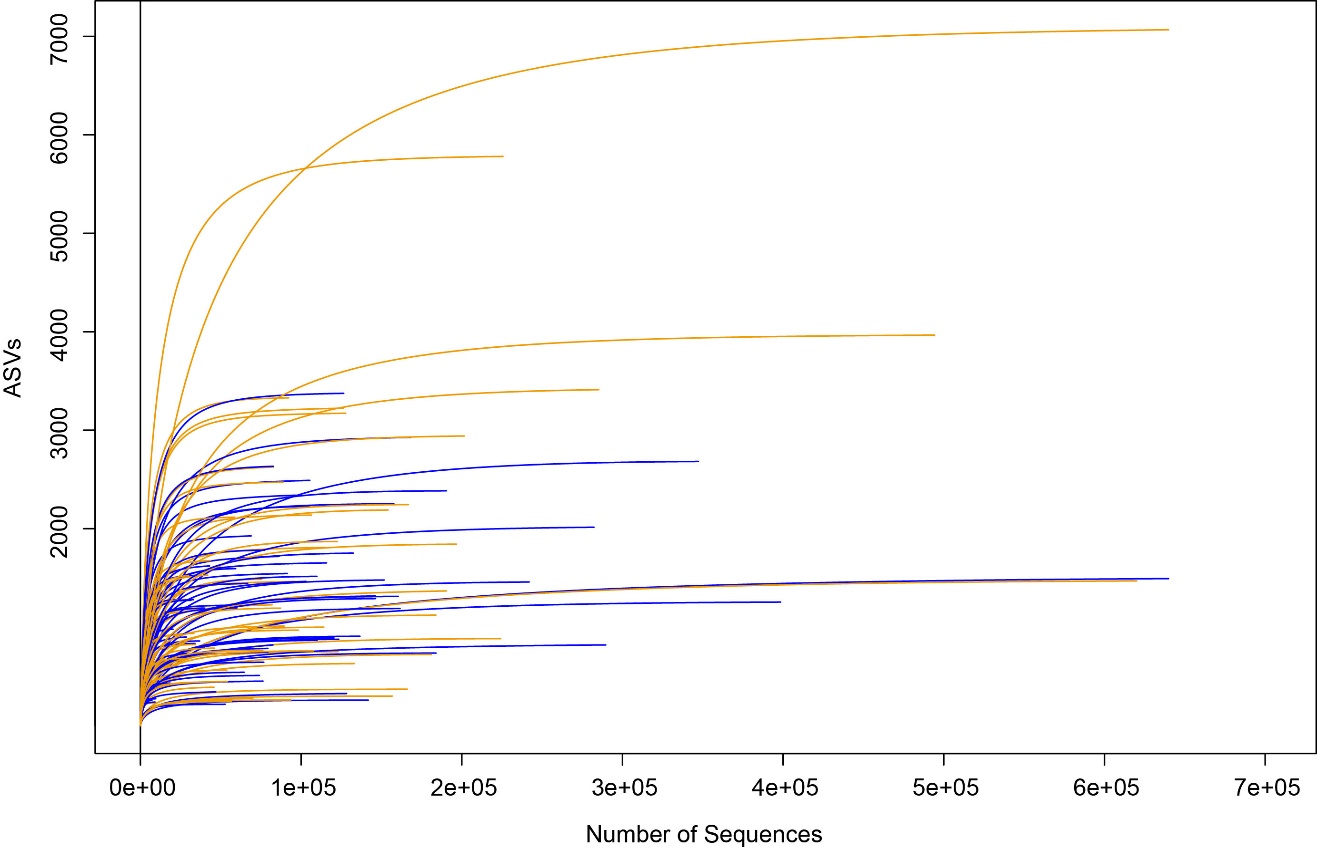
**

Fig. S13. Rarefaction curves. Sequence depth of each sample showing coverage of the diversity in each sampling site and each temperature within the gradient (6-35 °C). The y-axis indicates the number of ASVs detected while x-axis shows the number of sequences.

Supplementary Tables Information

Table S1. Statistical overview. Linear regression model analysis of the environmental variables in bottom water and surface sediment as well as bacterial production (comparing bays and incubation days), alpha diversity, VIF (variance inflation factor), and permutation analysis of the environmental variables and Bray-Curtis dissimilarities within a db-RDA ordination.

Table S2. Summary of selected environmental variables with mean±s.d.

Table S3. Bacterial production. Overview about the bacterial production data for each sampling site and incubated temperature after three, six, and nine days of incubation (plus field data).

Table S4. Sample overview. Shown are the sample names and associated environmental variables measured as well as the sampling date, sampling site, Shannon’s H, and Shannon’s H evenness. Furthermore, an overview of the metatranscriptomic samples with sampling date and sampling site are shown. Additionally, data of the oxygen, pH, and DIC (dissolved inorganic carbon) concentration in the bottom water of the incubated cores at different time points during the incubation are listed. Finally, an overview figure is included giving the temperatures tracked over the incubation time within the water bath.

Table S5. Sequencing information. Overview about the 16S rRNA gene amplicon Illumina sequencing with sample names, sampling date, DNA concentration (ng/µL), read length plus sequencing platform, sequenced reads per sample per million, and reads before and after quality filtering and chimera removal (final ASVs). Furthermore, an overview of the metatranscriptomic samples, their project ID from JGI, concentration (ng/µL), sampling date, sequencing platform and instrument, read length of the reads, as well as read count before and after filtering. Additionally, shown are the % of merged read pairs and the total amount of rRNA found and sequencing file name.

Table S6. Relative abundance of sequenced 16S rRNA ASVs. Overview of the sequenced samples with counts normalized to relative abundance per samples. Furthermore, taxonomical annotation for each ASV on kingdom, phylum, class, order, family, genus, and species levels are shown.

Table S7. Differential abundance analysis of the 16S rRNA gene amplicons. Overview of the relative abundance of the ASVs (relative abundances sum up to 1) that were significant for differential abundance analysis comparing field and incubated samples for bottom water and surface sediment. Additionally, an overview of the annotation of the ASVs up to species level are listed.

Table S8. Significant differential expressed transcripts with LFC >5. Overview of the significant differential expressed ORFs in the heated and control bay at 8, 16, and 28 °C with a log fold change of >5 (or <-5). Shown are ORFs, base mean, log2 fold change, standard error, statistics, *p* value, adjusted *p* value, sample name, the selected category used for analysis, taxonomic annotation up until species, different metabolic levels according to KEGG categories, and the associated gene and gene information.

Table S1. Overview about the used statistical approaches.

Table S1.1. Bottom water environmental variables. ANOVA of the chosen linear regression model (‘lm’ function within the ‘stats’ package in R) to test for differences in environmental parameters measured between bays and over the temperature gradient. Variable Bay indicates heated or control bay; Temp. includes the linear temperature gradient as a continuous variables from 6-35°C; Temp.sq. (temperature square) includes the curve linear temperature gradient; Bay/Site variables includes the sampling sites nested in the bay; and Variables separated by ‘:’ shows interaction effects tested within the model. Significance: ***, *p*<0.001; **, *p*<0.01; and *, p<0.05.

| Response variable | Variable | Df | Sum Sq. | Mean Sq. | F-value | *p*-value |
| --- | --- | --- | --- | --- | --- | --- |
| Sulfate | Bay | 1 | 0.218 | 0.218 | 0.607 | 0.440 |
|  | Temp. | 1 | 1.088 | 1.088 | 3.025 | 0.090 |
|  | Temp.sq. | 1 | 0.120 | 0.120 | 0.334 | 0.566 |
|  | Bay/Site | 4 | 2.114 | 0.528 | 1.469 | 0.230 |
|  | Bay:Temp. | 1 | 0.477 | 0.477 | 1.326 | 0.256 |
|  | Bay:Temp.Sq. | 1 | 0.308 | 0.308 | 0.858 | 0.360 |
| Salinity | Bay | 1 | 0.035 | 0.035 | 3.585 | 6.59E-02 |
|  | Temp. | 1 | 1.363 | 1.363 | 138.872 | 2.95E-14*** |
|  | Temp.sq. | 1 | 0.003 | 0.003 | 0.402 | 5.30E-01 |
|  | Bay/Site | 4 | 0.388 | 0.097 | 9.886 | 1.39E-05*** |
|  | Bay:Temp. | 1 | 0.040 | 0.040 | 4.095 | 5.01E-02 |
|  | Bay:Temp.Sq. | 1 | 0.0002 | 0.0002 | 0.023 | 8.80E-01 |
| Oxygen (Start) | Bay | 1 | 2.85E-03 | 2.85E-03 | 5.41E-03 | 9.42E-01 |
|  | Temp. | 1 | 115.025 | 115.025 | 218.292 | 2.46E-17*** |
|  | Temp.sq. | 1 | 1.97 | 1.97 | 3.74 | 6.07E-02 |
|  | Bay/Site | 4 | 1.74E-01 | 4.35E-02 | 8.25E-02 | 9.87E-01 |
|  | Bay:Temp. | 1 | 7.17E-02 | 7.17E-02 | 1.36E-01 | 7.14E-01 |
|  | Bay:Temp.Sq. | 1 | 1.62E-02 | 1.62E-02 | 3.08E-02 | 8.62E-01 |
| Ammonium | Bay | 1 | 15.623 | 15.623 | 0.007 | 0.932 |
|  | Temp. | 1 | 22826.922 | 22826.922 | 10.723 | 0.002*** |
|  | Temp.sq. | 1 | 37.231 | 37.231 | 0.017 | 0.895 |
|  | Bay/Site | 4 | 10136.071 | 2534.017 | 1.190 | 0.330 |
|  | Bay:Temp. | 1 | 30193.106 | 30193.106 | 14.183 | 0.0005*** |
|  | Bay:Temp.Sq. | 1 | 37275.447 | 37275.447 | 17.510 | 0.0001*** |
| pH | Bay | 1 | 8.53E-03 | 8.53E-03 | 0.141 | 0.709 |
|  | Temp. | 1 | 8.21E-02 | 8.21E-02 | 1.356 | 0.251 |
|  | Temp.sq. | 1 | 1.76E-05 | 1.76E-05 | 0.0002 | 0.986 |
|  | Bay/Site | 4 | 6.47E-01 | 1.62E-01 | 2.672 | 0.046* |
|  | Bay:Temp. | 1 | 3.99E-01 | 3.99E-01 | 6.590 | 0.014** |
|  | Bay:Temp.Sq. | 1 | 1.45E-01 | 1.45E-01 | 2.404 | 0.129 |
| Total Iron | Bay | 1 | 0.818 | 0.818 | 9.854 | 0.003** |
|  | Temp. | 1 | 0.610 | 0.610 | 7.346 | 0.010** |
|  | Temp.sq. | 1 | 0.062 | 0.062 | 0.751 | 0.391 |
|  | Bay/Site | 4 | 0.353 | 0.088 | 1.064 | 0.387 |
|  | Bay:Temp. | 1 | 0.330 | 0.330 | 3.973 | 0.053* |
|  | Bay:Temp.Sq. | 1 | 0.003 | 0.003 | 0.038 | 0.846 |
| Iron II | Bay | 1 | 1.03E-04 | 1.03E-04 | 0.01 | 0.898 |
|  | Temp. | 1 | 4.90E-02 | 4.90E-02 | 7.842 | 0.007** |
|  | Temp.sq. | 1 | 2.12E-05 | 2.12E-05 | 0.003 | 0.953 |
|  | Bay/Site | 4 | 1.32E-02 | 3.31E-03 | 0.529 | 0.714 |
|  | Bay:Temp. | 1 | 3.93E-04 | 3.93E-04 | 0.062 | 0.803 |
|  | Bay:Temp.Sq. | 1 | 1.46E-03 | 1.46E-03 | 0.232 | 0.632 |
| Nitrate/Nitrite (in combination) | Bay | 1 | 0.081 | 0.081 | 0.034 | 0.854 |
|  | Temp. | 1 | 19.498 | 19.498 | 8.220 | 0.006** |
|  | Temp.sq. | 1 | 0.155 | 0.155 | 0.065 | 0.799 |
|  | Bay/Site | 4 | 84.851 | 21.212 | 8.943 | 3.428E-05*** |
|  | Bay:Temp. | 1 | 0.140 | 0.140 | 0.059 | 0.808 |
|  | Bay:Temp.Sq. | 1 | 13.491 | 13.491 | 5.687 | 0.022* |
| Phosphate | Bay | 1 | 199.331 | 199.331 | 8.0289 | 7.33E-03** |
|  | Temp. | 1 | 1231.782 | 1231.782 | 49.615 | 2.15E-08*** |
|  | Temp.sq. | 1 | 625.896 | 625.896 | 25.210 | 1.24E-05*** |
|  | Bay/Site | 4 | 354.681 | 88.670 | 3.571 | 1.44E-02* |
|  | Bay:Temp. | 1 | 247.356 | 247.356 | 9.963 | 3.12E-03** |
|  | Bay:Temp.Sq. | 1 | 53.117 | 53.117 | 2.139 | 1.52E-01 |
| DC | Bay | 1 | 2367.425 | 2367.425 | 10.192 | 2.83E-03** |
|  | Temp. | 1 | 227.474 | 227.474 | 0.979 | 3.29E-01 |
|  | Temp.sq. | 1 | 13.741 | 13.741 | 0.059 | 8.09E-01 |
|  | Bay/Site | 4 | 13414.294 | 3353.573 | 14.437 | 2.95E-07*** |
|  | Bay:Temp. | 1 | 325.037 | 325.037 | 1.399 | 2.44E-01 |
|  | Bay:Temp.Sq. | 1 | 2731.918 | 2731.918 | 11.761 | 1.47E-03** |
| DOC | Bay | 1 | 4916.700 | 4916.700 | 12.949 | 0.0009*** |
|  | Temp. | 1 | 426.435 | 426.435 | 1.123 | 0.295 |
|  | Temp.sq. | 1 | 513.796 | 513.796 | 1.353 | 0.251 |
|  | Bay/Site | 4 | 9970.614 | 2492.653 | 6.565 | 0.0004*** |
|  | Bay:Temp. | 1 | 1.790 | 1.790 | 0.004 | 0.945 |
|  | Bay:Temp.Sq. | 1 | 1285.393 | 1285.393 | 3.385 | 0.073 |
| DIC | Bay | 1 | 358.777 | 358.777 | 3.180 | 0.082 |
|  | Temp. | 1 | 57.429 | 57.429 | 0.509 | 0.479 |
|  | Temp.sq. | 1 | 397.100 | 397.100 | 3.519 | 0.068 |
|  | Bay/Site | 4 | 913.674 | 228.418 | 2.024 | 0.110 |
|  | Bay:Temp. | 1 | 304.604 | 304.604 | 2.700 | 0.108 |
|  | Bay:Temp.Sq. | 1 | 233.414 | 233.414 | 2.069 | 0.158 |

Table 1.2. Porewater & sediment environmental variables. ANOVA of the chosen linear regression model (‘lm’ function within the ‘stats’ package in R) to test for differences in environmental parameters measured between bays and over the temperature gradient. Variable Bay indicates heated or control bay; Temp. includes the linear temperature gradient as a continuous variables from 6-35°C; Temp.sq. (temperature square) includes the curve linear temperature gradient; Bay/Site variables includes the sampling sites nested in the bay; Variables separated by ‘:’ shows interaction effects tested within the model.

| Response variable | Variable | Df | Sum Sq. | Mean Sq. | F-value | *p*-value |
| --- | --- | --- | --- | --- | --- | --- |
| Ammonium | Bay | 1 | 2413285.12 | 2413285.12 | 88.535 | 1.80E-11*** |
|  | Temp. | 1 | 268741.06 | 268741.06 | 9.859 | 3.26E-03** |
|  | Temp.sq. | 1 | 49203.23 | 49203.23 | 1.805 | 1.87E-01 |
|  | Bay/Site | 4 | 4484402.12 | 1121100.53 | 41.129 | 2.56E-13*** |
|  | Bay:Temp. | 1 | 46189.02 | 46189.02 | 1.694 | 2.01E-01 |
|  | Bay:Temp.Sq. | 1 | 23628.44 | 23628.44 | 0.866 | 3.58E-01 |
| pH | Bay | 1 | 0.002 | 0.002 | 0.056 | 0.813 |
|  | Temp. | 1 | 0.069 | 0.069 | 1.543 | 0.221 |
|  | Temp.sq. | 1 | 0.021 | 0.021 | 0.480 | 0.492 |
|  | Bay/Site | 4 | 0.712 | 0.178 | 3.976 | 0.008** |
|  | Bay:Temp. | 1 | 0.791 | 0.791 | 17.670 | 0.0001*** |
|  | Bay:Temp.Sq. | 1 | 0.0006 | 0.0006 | 0.014 | 0.905 |
| Sulfate | Bay | 1 | 0.862 | 0.862 | 12.937 | 9.15E-04*** |
|  | Temp. | 1 | 5.137 | 5.137 | 77.079 | 1.12E-10*** |
|  | Temp.sq. | 1 | 0.007 | 0.007 | 0.114 | 7.37E-01 |
|  | Bay/Site | 4 | 1.147 | 0.286 | 4.303 | 5.72E-03** |
|  | Bay:Temp. | 1 | 0.024 | 0.024 | 0.360 | 5.52E-01 |
|  | Bay:Temp.Sq. | 1 | 0.074 | 0.074 | 1.117 | 2.97E-01 |
| Total Iron | Bay | 1 | 487.136 | 487.136 | 4.411 | 0.042* |
|  | Temp. | 1 | 180.975 | 180.975 | 1.638 | 0.208 |
|  | Temp.sq. | 1 | 185.535 | 185.535 | 1.680 | 0.202 |
|  | Bay/Site | 4 | 1251.715 | 312.929 | 2.833 | 0.037* |
|  | Bay:Temp. | 1 | 490.023 | 490.023 | 4.437 | 0.041* |
|  | Bay:Temp.Sq. | 1 | 253.386 | 26 | 2.294 | 0.138 |
| Iron II | Bay | 1 | 15.459 | 15.459 | 6.024 | 1.88E-02* |
|  | Temp. | 1 | 18.818 | 18.818 | 7.333 | 1.01E-02* |
|  | Temp.sq. | 1 | 2.869 | 2.869 | 1.118 | 2.97E-01 |
|  | Bay/Site | 4 | 195.225 | 48.806 | 19.018 | 1.16E-08*** |
|  | Bay:Temp. | 1 | 4.099 | 4.099 | 1.597 | 2.14E-01 |
|  | Bay:Temp.Sq. | 1 | 0.121 | 0.121 | 0.047 | 8.29E-01 |
| Nitrate | Bay | 1 | 9595.292 | 9595.292 | 95.129 | 6.80E-12*** |
|  | Temp. | 1 | 31.926 | 31.926 | 0.316 | 5.77E-01 |
|  | Temp.sq. | 1 | 23.945 | 23.945 | 0.237 | 6.29E-01 |
|  | Bay/Site | 4 | 9235.645 | 2308.911 | 22.891 | 1.09E-09*** |
|  | Bay:Temp. | 1 | 374.976 | 374.976 | 3.717 | 6.13E-02 |
|  | Bay:Temp.Sq. | 1 | 83.235 | 83.235 | 0.825 | 3.69E-01 |
| Nitrite | Bay | 1 | 1.695 | 1.695 | 3.603 | 6.53E-02 |
|  | Temp. | 1 | 4.680 | 4.680 | 9.949 | 3.14E-03** |
|  | Temp.sq. | 1 | 0.052 | 0.052 | 0.110 | 7.41E-01 |
|  | Bay/Site | 4 | 48.229 | 12.057 | 25.630 | 2.40E-10*** |
|  | Bay:Temp. | 1 | 0.013 | 0.013 | 0.028 | 8.67E-01 |
|  | Bay:Temp.Sq. | 1 | 1.664 | 1.664 | 3.539 | 6.76E-02 |
| Phosphate | Bay | 1 | 110304.187 | 110304.187 | 69.753 | 3.958E-10*** |
|  | Temp. | 1 | 54862.536 | 54862.536 | 34.693 | 8.054E-07*** |
|  | Temp.sq. | 1 | 14.321 | 14.321 | 0.009 | 0.924 |
|  | Bay/Site | 4 | 706353.312 | 176588.328 | 111.670 | 1.818E-20*** |
|  | Bay:Temp. | 1 | 18228.638 | 18228.638 | 11.527 | 0.001** |
|  | Bay:Temp.Sq. | 1 | 49.945 | 49.945 | 0.031 | 0.859 |
| OM | Bay | 1 | 38.199 | 38.199 | 1.232 | 2.74E-01 |
|  | Temp. | 1 | 31.465 | 31.465 | 1.015 | 3.20E-01 |
|  | Temp.sq. | 1 | 10.679 | 10.679 | 0.344 | 5.61E-01 |
|  | Bay/Site | 4 | 1534.065 | 383.516 | 12.373 | 1.54E-06*** |
|  | Bay:Temp. | 1 | 24.982 | 24.982 | 0.805 | 3.75E-01 |
|  | Bay:Temp.Sq. | 1 | 1.129 | 1.129 | 0.036 | 8.50E-01 |
| DC | Bay | 1 | 45700.57 | 45700.57 | 3.603 | 0.065 |
|  | Temp. | 1 | 27495.72 | 27495.72 | 2.168 | 0.149 |
|  | Temp.sq. | 1 | 2886.38 | 2886.38 | 0.227 | 0.636 |
|  | Bay/Site | 4 | 197346.44 | 49336.61 | 3.890 | 0.009** |
|  | Bay:Temp. | 1 | 140668.59 | 140668.59 | 11.091 | 0.001*** |
|  | Bay:Temp.Sq. | 1 | 28465.32 | 28465.32 | 2.244 | 0.142 |
| DOC | Bay | 1 | 58127.832 | 58127.832 | 6.681 | 0.013* |
|  | Temp. | 1 | 17935.991 | 17935.991 | 2.061 | 0.159 |
|  | Temp.sq. | 1 | 338.525 | 338.525 | 0.038 | 0.844 |
|  | Bay/Site | 4 | 189120.705 | 47280.176 | 5.434 | 0.001*** |
|  | Bay:Temp. | 1 | 93949.795 | 93949.795 | 10.798 | 0.002** |
|  | Bay:Temp.Sq. | 1 | 11348.504 | 11348.504 | 1.304 | 0.260 |
| DIC | Bay | 1 | 703.035 | 703.035 | 2.769 | 0.104 |
|  | Temp. | 1 | 0.332 | 0.332 | 0.001 | 0.971 |
|  | Temp.sq. | 1 | 41.498 | 41.498 | 0.163 | 0.688 |
|  | Bay/Site | 4 | 3029.796 | 757.449 | 2.984 | 0.030* |
|  | Bay:Temp. | 1 | 962.008 | 962.008 | 3.790 | 0.058 |
|  | Bay:Temp.Sq. | 1 | 222.408 | 222.408 | 0.876 | 0.355 |

^***^: p<0.001 ^**^: p<0.01 ^*^: p<0.05

Table S1.3. Bacterial production. ANOVA of chosen linear regression model (‘lm’ function within the ‘stats’ package in R) to test for differences in bacterial production between bays and over the temperature gradient and incubation time. Variable Bay indicates heated or control bay; Time includes the time points the samples were taken at 3, 6, and 9 days of incubation; Temp. estimates the linear relationship with incubation temperature (treated as a continuous variable from 6-35 °C); Temp.sq. (temperature square) estimates the curvilinear (quadratic) relationship with temperature; Bay/Site variables includes the sampling sites nested in the bay; Variables separated by ‘:’ shows interaction effects tested within the model.

| Variable | Df | Sum Sq. | Mean Sq. | F-value | *p*-value |
| --- | --- | --- | --- | --- | --- |
| Bay | 1 | 14556.5707 | 14556.5707 | 7.78E-01 | 3.80E-01 |
| Time | 2 | 4223870.727 | 2111935.364 | 1.13E+02 | 2.19E-28*** |
| Temp. | 1 | 1683356.608 | 1683356.608 | 9.00E+01 | 2.73E-16*** |
| Temp.sq. | 1 | 182.2157 | 182.2157 | 9.74E-03 | 9.22E-01 |
| Bay/Site | 4 | 195658.2526 | 48914.5632 | 2.61E+00 | 3.86E-02* |
| Time:Temp. | 2 | 799249.765 | 399624.8825 | 2.14E+01 | 1.14E-08*** |
| Bay:Time | 2 | 736197.7557 | 368098.8779 | 1.97E+01 | 4.01E-08*** |
| Bay:Temp. | 1 | 108581.2801 | 108581.2801 | 5.80E+00 | 1.75E-02* |
| Bay:Temp.sq. | 1 | 604.9263 | 604.9263 | 3.23E-02 | 8.58E-01 |
| Time:Temp.sq. | 2 | 12292.7665 | 6146.3832 | 3.28E-01 | 7.21E-01 |
| Bay:Time:Temp. | 2 | 132678.9717 | 66339.4858 | 3.54E+00 | 3.19E-02* |
| Bay:Time:Temp.sq. | 2 | 69590.7304 | 34795.3652 | 1.86E+00 | 1.60E-01 |

^***^: p<0.001 ^**^: p<0.01 ^*^: p<0.05

Table S1.4. Bacterial production test for each Bay along the temperature gradient and time. ANOVA of chosen linear regression model (‘lm’ function within the ‘stats’ package in R) to test for differences in bacterial production in each bay over the temperature gradient and incubation time. Time includes the time points the samples were taken at 3, 6, and 9 days of incubation; Temp. estimates the linear relationship with incubation temperature (treated as a continuous variable from 6-35 °C); Temp.sq. (temperature square) estimates the curvilinear (quadratic) relationship with temperature; Site variables includes the sampling sites in the bays; Variables separated by ‘:’ shows interaction effects tested within the model.

| Bay | Variable | Df | Sum Sq. | Mean Sq | F-value | *p*-value |
| --- | --- | --- | --- | --- | --- | --- |
| Heated | Site | 2 | 129526.300 | 64763.130 | 2.679 | 7.64E-02 |
|  | Time | 2 | 2669469.000 | 1334735.000 | 55.217 | 1.40E-14*** |
|  | Temp. | 1 | 468103.800 | 468103.800 | 19.365 | 4.25E-05*** |
|  | Temp.Sq. | 1 | 52.049 | 52.049 | 0.002 | 9.63E-01 |
|  | Time:Temp. | 2 | 242644.200 | 121322.100 | 5.019 | 9.49E-03** |
| Control | Site | 2 | 78157.034 | 39078.517 | 2.942 | 6.01E-02 |
|  | Time | 2 | 2271049.865 | 1135524.933 | 85.502 | 1.50E-18*** |
|  | Temp. | 1 | 1334964.773 | 1334964.773 | 100.519 | 1.35E-14*** |
|  | Temp.Sq. | 1 | 596.912 | 596.912 | 0.045 | 8.33E-01 |
|  | Time:Temp. | 2 | 685824.356 | 342912.178 | 25.820 | 6.96E-09*** |

^***^: p<0.001 ^**^: p<0.01 ^*^: p<0.05

Table S1.4.1. Pairwise comparison of different sampling time points for bacterial production for each bay along the temperature gradient. Function emmeans was used to do a pairwise comparison on the used model from Table S2.4 between the different sampling time points at 3, 6, and 9 days of incubation for each bay. Contrast (time) was used to compare each time point by temperature (continuous variable). Estimate shows the estimate of the effect size; Df shows the degrees of freedom; t.ratio shows the test statistic used to compute the p-value.

| Bay | Contrast | Temp. | Estimate | SE | Df | T.ratio | *p*-value |
| --- | --- | --- | --- | --- | --- | --- | --- |
| Heated | T3 - T6 | 19.625 | -471.463 | 44.882 | 63 | -10.505 | 1.53E-11*** |
|  | T3 - T9 | 19.625 | -224.148 | 44.882 | 63 | -4.994 | 1.47E-05*** |
|  | T6 - T9 | 19.625 | 247.315 | 44.882 | 63 | 5.510 | 2.11E-06*** |
| Control | T3 - T6 | 19.507 | -318.655 | 33.646 | 62 | -9.471 | 1.93E-11*** |
|  | T3 - T9 | 19.507 | -419.586 | 33.646 | 62 | -12.471 | 1.89E-11*** |
|  | T6 - T9 | 19.507 | -100.931 | 33.270 | 62 | -3.034 | 9.74E-03** |

^***^: p<0.001 ^**^: p<0.01 ^*^: p<0.05

**Table S1.5. Bacterial production test for each time point comparing bays.** ANOVA of chosen linear regression model (‘lm’ function within the ‘stats’ package in R) to test for differences in bacterial production in each bay over the temperature gradient. Temp. includes the linear temperature gradient as a continuous variables from 6-35 °C; Temp.sq. (temperature square) includes the curve linear temperature gradient; Site variables includes the sampling sites in the bays; Variables separated by ‘:’ shows interaction effects tested within the model.

| Time | Variable | Df | Sum Sq. | Mean Sq. | F-value | *p*-value |
| --- | --- | --- | --- | --- | --- | --- |
| Day 3 | Bay | 1 | 1.133 | 1.133 | 0.198 | 6.59E-01 |
|  | Temp. | 1 | 153.353 | 153.353 | 26.763 | 8.25E-06*** |
|  | Temp.Sq. | 1 | 39.571 | 39.571 | 6.906 | 1.24E-02* |
|  | Bay/Site | 4 | 22.638 | 5.659 | 0.988 | 4.26E-01 |
|  | Bay:Temp. | 1 | 0.217 | 0.217 | 0.038 | 8.47E-01 |
|  | Bay:Temp.Sq. | 1 | 3.395 | 3.395 | 0.592 | 4.46E-01 |
| Day 6 | Bay | 1 | 268002.707 | 268002.707 | 8.312 | 6.45E-03** |
|  | Temp. | 1 | 1095941.985 | 1095941.985 | 33.992 | 9.73E-07*** |
|  | Temp.Sq. | 1 | 4590.965 | 4590.965 | 0.142 | 7.08E-01 |
|  | Bay/Site | 4 | 134745.388 | 33686.347 | 1.045 | 3.97E-01 |
|  | Bay:Temp. | 1 | 6210.487 | 6210.487 | 0.193 | 6.63E-01 |
|  | Bay:Temp.Sq. | 1 | 43825.178 | 43825.178 | 1.359 | 2.51E-01 |
| Day 9 | Bay | 1 | 478930.596 | 478930.596 | 19.540 | 7.96E-05** |
|  | Temp. | 1 | 1393846.711 | 1393846.711 | 56.868 | 4.61E-09*** |
|  | Temp.Sq. | 1 | 7894.302 | 7894.302 | 0.322 | 5.74E-01 |
|  | Bay/Site | 4 | 160512.634 | 40128.159 | 1.637 | 1.85E-01 |
|  | Bay:Temp. | 1 | 234999.476 | 234999.476 | 9.588 | 3.67E-03** |
|  | Bay:Temp.Sq. | 1 | 26387.333 | 26387.333 | 1.077 | 3.06E-01 |

^***^: p<0.001 ^**^: p<0.01 ^*^: p<0.05

Table S1.6. Alpha Diversity in bottom water communities. ANOVA of chosen linear regression model (‘lm’ function within the ‘stats’ package in R) to test for differences in alpha diversity between bays and over the temperature gradient. Variable Bay indicates heated or control bay; Temp. includes the linear temperature gradient as a continuous variable from 6-35 °C; Temp.sq. (temperature square) includes the curve linear temperature gradient; Bay/Site variables includes the sampling sites nested in the bay; Variables separated by ‘:’ shows interaction effects tested within the model.

| Response variable | Variable | Df | Sum Sq. | Mean Sq. | F-value | *p*-value |
| --- | --- | --- | --- | --- | --- | --- |
| Shannon’s H | Bay | 1 | 0.240 | 0.240 | 0.237 | 0.627 |
|  | Temp. | 1 | 27.064 | 27.064 | 26.783 | 3.186E-06*** |
|  | Temp.sq. | 1 | 1.693 | 1.693 | 1.675 | 0.200 |
|  | Bay/Site | 4 | 12.946 | 3.234 | 3.202 | 0.019* |
|  | Bay:Temp, | 1 | 0.963 | 0.962 | 0.953 | 0.333 |
|  | Bay:Temp.sq. | 1 | 2.475 | 1.010 | 2.448 | 0.123 |
| Shannon’s H evenness | Bay | 1 | 2.00E-05 | 2.00E-05 | 0.001 | 9.73E-01 |
|  | Temp. | 1 | 0.377 | 0.377 | 23.135 | 1.17E-05*** |
|  | Temp.sq. | 1 | 0.005 | 0.005 | 0.347 | 0.557 |
|  | Bay/Site | 4 | 0.282 | 0.070 | 4.333 | 0.004** |
|  | Bay:Temp, | 1 | 0.014 | 0.001 | 0.122 | 0.728 |
|  | Bay:Temp.sq. | 1 | 0.912 | 0.014 | 0.879 | 0.352 |

^***^: p<0.001 ^**^: p<0.01 ^*^: p<0.05

Table S1.7. Alpha diversity in surface sediment communities. ANOVA of chosen linear regression model (‘lm’ function within the ‘stats’ package in R) to test for differences in alpha diversity between bays and over the temperature gradient. Variable Bay indicates heated or control bay; Temp. includes the linear temperature gradient as a continuous variable from 6-35 °C; Temp.sq. (temperature square) includes the curve linear temperature gradient; Bay/Site variables includes the sampling sites nested in the bay; Variables separated by ‘:’ shows interaction effects tested within the model.

| Response variable | Variable | Df | Sum Sq. | Mean Sq. | F-value | *p*-value |
| --- | --- | --- | --- | --- | --- | --- |
| Shannon’s H | Bay | 1 | 5.23E-03 | 0.005 | 0.017 | 0.894 |
|  | Temp. | 1 | 0.133 | 0.133 | 0.456 | 0.501 |
|  | Temp.sq. | 1 | 0.425 | 0.425 | 1.456 | 0.232 |
|  | Bay/Site | 4 | 17.632 | 4.408 | 15.082 | 2.20E-08*** |
|  | Bay:Temp, | 1 | 0.192 | 0.192 | 0.659 | 0.420 |
|  | Bay:Temp.sq. | 1 | 0.313 | 0.313 | 1.072 | 0.305 |
| Shannon’s H evenness | Bay | 1 | 0.002 | 0.002 | 6.934 | 0.010* |
|  | Temp. | 1 | 0.0001 | 0.0001 | 0.291 | 0.591 |
|  | Temp.sq. | 1 | 0.0003 | 0.0003 | 0.9227 | 0.340 |
|  | Bay/Site | 4 | 0.034 | 0.008 | 22.727 | 4.03E-11*** |
|  | Bay:Temp, | 1 | 0.0009 | 0.0009 | 2.628 | 0.110 |
|  | Bay:Temp.sq. | 1 | 0.020 | 0.0003 | 0.710 | 0.402 |

^***^: p<0.001 ^**^: p<0.01 ^*^: p<0.05

Table S1.8. Variance inflation factor (VIF) for bottom water communities. Test if environmental variables add explanatory information to the differences between bacterial communities using db-RDA (based on Bray-Curtis dissimilarities). A VIF<5 indicates that the variable explains part of the differences between bacterial communities of the two bays and different temperatures.

| Environmental variable | VIF |
| --- | --- |
| Oxygen | 1.67 |
| pH | 1.73 |
| Sulfate | 1.23 |
| Iron II | 2.58 |
| Ammonium | 1.61 |
| Nitrate/Nitrite (in combination) | 1.42 |
| Salinity | 1.62 |
| Phosphate | 1.97 |
| Depth | 1.48 |

Table S1.9. Variance inflation factor (VIF) for surface sediment communities. Test if environmental variables add explanatory information to the differences between bacterial communities using db-RDA (based on Bray-Curtis dissimilarities). A VIF<5 indicates that the variable explains part of the differences between bacterial communities of the two bays and different temperatures.

| Environmental variable | VIF |
| --- | --- |
| OM | 1.51 |
| pH | 3.55 |
| Iron II | 1.91 |
| Sulfate | 3.86 |
| Ammonium | 4.42 |
| Nitrate | 4.35 |
| Phosphate | 5.08 |
| Depth | 1.51 |

Table S1.10. ANOVA test for dbRDA under direct model with marginal effects of terms within each bay to test for effects within the temperature gradient in bottom water bacterial communities. Number of permutations *n*=999. Test of which environmental variables significantly explain variation on bacterial communities in response of the temperature in each bay.

| Variables | Df | Sum Sq. | F-value | *p*-value |
| --- | --- | --- | --- | --- |
| Oxygen | 1 | 0.933 | 2.717 | 0.001*** |
| pH | 1 | 0.473 | 1.378 | 0.112 |
| Sulfate | 1 | 0.360 | 1.050 | 0.376 |
| Iron II | 1 | 0.565 | 1.645 | 0.041* |
| Ammonium | 1 | 0.640 | 1.864 | 0.015* |
| Nitrate/Nitrite (in combination) | 1 | 0.227 | 0.662 | 0.915 |
| Salinity | 1 | 0.372 | 1.083 | 0.342 |
| Phosphate | 1 | 0.467 | 1.361 | 0.102 |
| Depth | 1 | 0.301 | 0.878 | 0.603 |

^***^: p<0.001 ^**^: p<0.01 ^*^: p<0.05

Table S1.11. ANOVA test for dbRDA under direct model with marginal effects of terms within each bay to test for effects within the temperature gradient in surface sediment bacterial communities. Number of permutations *n*=999. Test of which environmental variables significantly explain variation on bacterial communities in response of the temperature in each bay.

| Variables | Df | Sum Sq. | F-value | *p*-value |
| --- | --- | --- | --- | --- |
| OM | 1 | 1.101 | 4.699 | 0.001*** |
| pH | 1 | 0.623 | 2.661 | 0.013* |
| Iron II | 1 | 0.493 | 2.103 | 0.046* |
| Sulfate | 1 | 0.574 | 2.451 | 0.013* |
| Ammonium | 1 | 0.268 | 1.146 | 0.285 |
| Nitrate | 1 | 0.228 | 0.974 | 0.493 |
| Phosphate | 1 | 0.435 | 1.860 | 0.056 |
| Depth | 1 | 0.188 | 0.802 | 0.788 |

^***^: p<0.001 ^**^: p<0.01 ^*^: p<0.05

**Table S2. Selected environmental variables mean and standard variation overview.**

| **Variable** | **Type** | **Bay** | **Temperature (°C)** | **mean** | **±s.d.** |
| --- | --- | --- | --- | --- | --- |
| pH | Bottom water | Control | 6 | 8.51 | 0.11 |
|  |  |  | 35 | 8.72 | 0.30 |
|  |  | Heated | 6 | 9.02 | 0.06 |
|  |  |  | 35 | 8.54 | 0.51 |
| Nitrate+Nitrite (in combination) | Bottom water | Control | 6 | 0.57 | 0.16 |
|  |  |  | 35 | 1.77 | 2.70 |
|  |  | Heated | 6 | 1.97 | 1.06 |
|  |  |  | 35 | 3.48 | 1.84 |
| Iron II | Bottom water | Control | 6 | 0.44 | 0.04 |
|  |  |  | 35 | 0.59 | 0.08 |
|  |  | Heated | 6 | 0.44 | 010 |
|  |  |  | 35 | 0.54 | 0.10 |
| Phosphate | Bottom water | Control | 6 | 0.52 | 0-28 |
|  |  |  | 35 | 27.30 | 16.86 |
|  |  | Heated | 6 | 0.55 | 0.68 |
|  |  |  | 35 | 13.05 | 9.12 |
| Total iron | Bottom water | Control | 6 | 1.03 | 0.10 |
|  |  |  | 35 | 1.50 | 0.35 |
|  |  | Heated | 6 | 1.07 | 0.14 |
|  |  |  | 35 | 1.00 | 0.04 |
| pH | Pore water | Control | 6 | 8.59 | 0.005 |
|  |  |  | 35 | 8.28 | 0.24 |
|  |  | Heated | 6 | 8.34 | 0.23 |
|  |  |  | 35 | 8.50 | 0.11 |
|  |  | Control | Field | 7.19 | 0.17 |
|  |  | Heated | Field | 7.21 | 0.15 |
| Ammonium | Pore Water | Control | Incubation (6-35) | 724.35 | 113.24 |
|  |  | Heated | Incubation (6-35) | 491.15 | 311.53 |
|  |  |  | Field | 240.99 | 180.56 |
| Iron II | Pore Water | Control | 6 | 4.97 | 0.73 |
|  |  |  | 35 | 6.39 | 2.96 |
|  |  | Heated | 6 | 3.55 | 3.33 |
|  |  |  | 35 | 5.33 | 3.17 |
| Phosphate | Pore Water | Control | 6 | 252.25 | 116.10 |
|  |  |  | 35 | 394.09 | 67.13 |
|  |  | Heated | 6 | 197.25 | 187.62 |
|  |  |  | 35 | 242.75 | 188.51 |
| Nitrate | Pore Water | Control | 6 | 80.31 | 30.64 |
|  |  |  | 35 | 68.86 | 5.12 |
|  |  | Heated | 6 | 39.40 | 7.82 |
|  |  |  | 35 | 46.17 | 4.03 |
|  |  |  | Field | 29.75 | 16.52 |
| Sulfate | Pore Water | Control | 6 | 3.82 | 0.14 |
|  |  |  | 35 | 2.61 | 0.17 |
|  |  | Heated | 6 | 3.49 | 0.19 |
|  |  |  | 35 | 2.44 | 0.19 |
| OM | Sediment | Control | Field | 30.30 | 2.19 |
|  |  | Heated | Field | 47.89 | 13.10 |

**References**

1. L. Seidel, E. Broman, M. Ståhle, E. Nilsson, S. Turner, W. Hendrycks et al., Long-term warming of Baltic Sea coastal waters affects bacterial communities in bottom water and sediments differently. Front. Microbiol. 13, 873281 (2022).

2. L. Seidel, M. Ketzer, E. Broman, S. Shahabi-Ghahfarokhi, M. Rahmati-Abkenar, S. Turner et al., Weakened resilience of benthic microbial communities in the face of climate change. ISME Commun. 2, 21 (2022).

3. J. C. Valderrama (2015) "Methods of nutrient analysis" in Manual of harmful marine microalgae*,* G. M. Hallegraeff, D. M. Anderson, A. D. Cembella, Eds. (Intergovernmental Oceanographic Commision of UNESCO, Paris, 1995), pp. 251-268.

4. L. W. Hugerth, H. A. Wefer, S. Lundin, H. E. Jakobsson, M. Lindberg, S. Rodin *et al.* (2014) DegePrime, a program for degenerate primer design for broad-taxonomic-range PCR in microbial ecology studies. *AEM*  **80**, 5116-5123.

5. M. V. Lindh, D. Figueroa, J. Sjöstedt, F. Baltar, D. Lundin, A. Andersson *et al.* (2015) Transplant experiments uncover Baltic Sea basin-specific responses in bacterioplankton community composition and metabolic activities. *Front. Microbiol.* **6**, 223.

6. D. Straub, N. Blackwell, A. Langarica-Fuentes, A. Peltzer, S. Nahnsen, S. Kleindienst (2020) Interpretations of environmental microbial community studies are biased by the selected 16S rRNA (gene) amplicon sequencing pipeline. *Front. Microbiol.* **11**, 550420.

7. R Core Team (2018) R: A language and environment for statistical computing. (R Foundation for statistical Computing, Vienna, Austria).

8. C. Reimann, P. Filzmoser, K. Fabian, K. Hron, M. Birke, A. Demetriades *et al.* (2012) The concept of compositional data analysis in practice--total major element concentrations in agricultural and grazing land soils of Europe. *Sci. Total Environ.* **426**, 196-210.

9. J. Oksanen *et al.* (2019) vegan: community ecology package. (CRAN.R-project.org/package=vegan).

10. L. Beule, P. Karlovsky (2020) Improved normalization of species count data in ecology by scaling with ranked subsampling (SRS): application to microbial communities. *Peer J* **8**, e9593.

11. R. V. Lenth (2021) emmeans: estimated marginal means, aka least-squares means. (CRAN.R-project.org/package=emmeans).

12. A. F. Zuur, E. N. Leno, C. S. Elphick (2010) A protocol for data exploration to avoid common statistical problems. *MEE* **1**, 3-14.

13. H. Wickham (2017) tidyverse: Easily Install and Load the "Tidyverse". (CRAN.R-project.org/package=tidyverse).

14. M. I. Love, W. Huber, S. Anders (2014) Moderated estimation of fold change and dispersion for RNA-seq data with DESeq2. *Genome Biol.* **15**, 550.

15. B. Bushnell (2014) BBMap. (Sourceforge.net/projects/bbmap/).

16. D. Li, C.-M. Liu, R. Luo, K. Sadakane, T.-W. Lam (2015) MEGAHIT: an ultra-fast single-node solution for large and complex metagenomics assembly via succinct de Bruijn graph. *Bioinformatics* **31**, 1674-1676.

17. D. Hyatt, G.-L. Chen, P. F. Locascio, M. L. Land, F. W. Larimer, L. J. Hauser et al., Prodigal: prokaryotic gene recognition and translation initiation site identification. BMC Bioinform. 11, 119 (2010).

18. C. P. Cantalapiedra, A. Hernández-Plaza, I. Letunic, P. Bork, J. Huerta-Cepas, eggNOG-mapper v2: functional annotation, orthology assignments, and domain prediction at the metagenomic scale. Mol. Biol. Evol. 38, 5825-5829 (2021).

19. J. Huerta-Cepas, D. Szklarczyk, D. Heller, A. Hernández-Plaza, S. K. Forslund, H. Cook et al., eggNOG 5.0: a hierarchical, functionally and phylogenetically annotated orthology resource based on 5090 organisms and 2502 viruses. Nucleic Acids Research 47, D309-D314 (2018).

20. A. Krinos, S. K. Hu, N. R. Cohen, H. Alexander, EUKulele: Taxonomic annotation of the unsung eukaryotic microbes. J. Open Source Softw. 6, https://arxiv.org/abs/2011.00089 (2021).

21. Y. Liao, G. K. Smyth, W. Shi, featureCounts: an efficient general purpose program for assigning sequence reads to genomic features. Bioinformatics 30, 923-930 (2013).

22. S. C. Goslee, D. L. Urban, The ecodist package for dissimilarity-based analysis of ecological data. J. Stat. Softw. 22, 1 - 19 (2007).

23. H. Wickham (2016) ggplot2: Elegant Graphics for Data Analysis. (ggplot2.tidyverse.org).
